# Morbidity, mortality, and long-term consequences associated with diarrhoea from *Cryptosporidium* infection in children younger than 5 years: a meta-analyses study

**DOI:** 10.1016/S2214-109X(18)30283-3

**Published:** 2018-06-13

**Authors:** Ibrahim A Khalil, Christopher Troeger, Puja C Rao, Brigette F Blacker, Alexandria Brown, Thomas G Brewer, Danny V Colombara, Eugenio L De Hostos, Cyril Engmann, Richard L Guerrant, Rashidul Haque, Eric R Houpt, Gagandeep Kang, Poonum S Korpe, Karen L Kotloff, Aldo A M Lima, William A Petri, James A Platts-Mills, David A Shoultz, Mohammed H Forouzanfar, Simon I Hay, Robert C Reiner, Ali H Mokdad

**Affiliations:** a Institute for Health Metrics and Evaluation, University of Washington, Seattle, WA, USA; b PATH, San Fransisco, CA, USA; c PATH, Seattle, WA, USA; d School of Public Health, University of Washington, Seattle, WA, USA; e Department of Global Health, University of Washington, Seattle, WA, USA; f Center for Global Health, Division of Infectious Diseases and International Health, University of Virginia, Charlottesville, VA, USA; g icddr,b, Dhaka, Bangladesh; h Translational Health Science and Technology Institute, Faridabad, India; i Department of Epidemiology, Johns Hopkins Bloomberg School of Public Health, Baltimore, MD, USA; j Departments of Pediatrics and Medicine, Center for Vaccine Development, Institute for Global Health, University of Maryland School of Medicine, Baltimore, MD, USA; k Center for Global Health, Federal University of Ceara, Fortaleza, Ceara, Brazil; l Big Data Institute, Li Ka Shing Centre for Health Information and Discovery, University of Oxford, Oxford, UK

## Abstract

**Background:**

The protozoan *Cryptosporidium* is a leading cause of diarrhoea morbidity and mortality in children younger than 5 years. However, the true global burden of *Cryptosporidium* infection in children younger than 5 years might have been underestimated in previous quantifications because it only took account of the acute effects of diarrhoea. We aimed to demonstrate whether there is a causal relation between *Cryptosporidium* and childhood growth and, if so, to quantify the associated additional burden.

**Methods:**

The Global Burden of Diseases, Injuries, and Risk Factors study (GBD) 2016 was a systematic and scientific effort to quantify the morbidity and mortality associated with more than 300 causes of death and disability, including diarrhoea caused by *Cryptosporidium* infection. We supplemented estimates on the burden of *Cryptosporidium* in GBD 2016 with findings from a systematic review of published and unpublished cohort studies and a meta-analysis of the effect of childhood diarrhoea caused by *Cryptosporidium* infection on physical growth.

**Findings:**

In 2016, *Cryptosporidium* infection was the fifth leading diarrhoeal aetiology in children younger than 5 years, and acute infection caused more than 48 000 deaths (95% uncertainty interval [UI] 24 600–81 900) and more than 4·2 million disability-adjusted life-years lost (95% UI 2·2 million–7·2 million). We identified seven data sources from the scientific literature and six individual-level data sources describing the relation between *Cryptosporidium* and childhood growth. Each episode of diarrhoea caused by *Cryptosporidium* infection was associated with a decrease in height-for-age *Z* score (0·049, 95% CI 0·014–0·080), weight-for-age *Z* score (0·095, 0·055–0·134), and weight-for-height *Z* score (0·126, 0·057–0·194). We estimated that diarrhoea from *Cryptosporidium* infection caused an additional 7·85 million disability-adjusted life-years (95% UI 5·42 million–10·11 million) after we accounted for its effect on growth faltering—153% more than that estimated from acute effects alone.

**Interpretation:**

Our findings show that the substantial short-term burden of diarrhoea from *Cryptosporidium* infection on childhood growth and wellbeing is an underestimate of the true burden. Interventions designed to prevent and effectively treat infection in children younger than 5 years will have enormous public health and social development impacts.

**Funding:**

The Bill & Melinda Gates Foundation.

## Introduction

*Cryptosporidium* is an intracellular protozoan parasite that was first recognised as a causative agent of diarrhoea in 1976.^1^ Invasive *Cryptosporidium* infection of the small intestine causes damage to the intestinal epithelium^2^ and disrupts absorption and barrier function,^3^ leading to mild-to-severe diarrhoea. *Cryptosporidium* is well adapted to infect human beings and animals through zoonotic, waterborne, foodborne, and human-to-human routes of transmission. These routes enable *Cryptosporidium* to be endemic in many low-income countries and potentially epidemic in high-income countries, including the USA.^4^, ^5^ *Cryptosporidium* species have a low infective threshold and are also highly resistant to many disinfectants such as chlorine.^4^ Many infections are asymptomatic or mild and self-limiting, and they often go unrecognised. The wide range of disease severity is affected by the host's age, the host's nutritional and immune status, and possibly by the infecting species and subtype.^6^ These adaptations have contributed to a substantial global burden of disease.

The consequences of *Cryptosporidium* infection in children might extend far beyond diarrhoeal episodes. Symptomatic and asymptomatic *Cryptosporidium* infections in children living in low-resource settings are sometimes associated with malnutrition and stunted growth.^7^, ^8^, ^9^, ^10^ This association might be mediated by environmental enteropathy or environmental enteric dysfunction, a broad intestinal syndrome characterised by local inflammation, nutrient malabsorption, barrier disruption, and bacterial translocation that is thought to be a result of chronic exposure to a variety of enteric pathogens (including protozoans such as *Cryptosporidium*).^11^ Malnutrition might predispose children to infection, but a *Cryptosporidium* infection could also impede nutrient absorption and thereby cause malnutrition, leading to a longer lasting and vicious cycle of reinfection.^12^, ^13^ The severity and extent of these outcomes warrant further study.

**Research in context**

**Evidence before this study**

In the Global Burden of Disease Study (GBD) 2015, diarrhoea was estimated to have caused 499 000 deaths (95% uncertainty interval 447 000–558 000), and *Cryptosporidium* was one of the three aetiologies responsible for the most deaths in children younger than 5 years. *Cryptosporidium* has long been recognised to be associated with long-term sequelae, including linear growth shortfalls (stunting). We searched PubMed on May 24, 2018, with the terms Cryptospor* AND (“stunting” OR “wasting” OR “growth” OR “malnutrition”) AND (“diarrhea”) AND (“prospective” OR “case control” OR “trial” OR “cohort”), without date or language restrictions and identified 103 publications. Beginning in the mid-1990s, an association between *Cryptosporidium* infection and childhood growth failure was identified in cohort studies in Brazil, Peru, and Guinea-Bissau. Although these publications suggested the potentially large impact of *Cryptosporidium* infection on weight and height gain, none of the studies in our search result attempted to synthesise all of the available data on *Cryptosporidium* infection and childhood growth or attempted to quantify the total burden of *Cryptosporidium* infection.

**Added value to this study**

We present findings from GBD 2016 and build on this work to evaluate a proposed causal relation between *Cryptosporidium* infection and childhood growth and to quantify the additional burden associated with non-fatal infection due to poor growth outcomes. Our findings suggest that the previously reported burden of *Cryptosporidium* infection is underestimated and that *Cryptosporidium* diarrhoea affects childhood health beyond acute illness by decreasing growth. By properly accounting for these long-term outcomes, we estimated that the burden of *Cryptosporidium* infection is 2·5 times larger than previously reported.

**Implications of all the available evidence**

The findings have large implications for global public health leaders. Our work provides valuable insight into the true burden of diarrhoea from *Cryptosporidium* infection and illuminates the challenges and the potential intervention strategies for addressing the global *Cryptosporidium* infection burden. Our interpretations highlight important knowledge gaps and research priorities, and we call for renewed efforts from policy makers and research funders to accelerate programmes and interventions that maximise health and long-term outcomes.

Differences in methods, type of diagnostics used, and study populations between studies have resulted in a large variation in burden estimates for diarrhoea from *Cryptosporidium* infection. Understanding the worldwide burden of *Cryptosporidium* infection and infection-related consequences is the first step toward determining the appropriate strategy for intervention. In the Global Enteric Multicenter Study (GEMS), *Cryptosporidium* was one of the four major contributors to moderate-to-severe diarrhoeal diseases during the first 2 years of life in all seven sites, and *Cryptosporidium* was a key pathogen in diarrhoeal disease.^14^ *Cryptosporidium* infection was also associated with more than a two-fold increase in the risk of death in children aged 12–23 months who were admitted to hospital with diarrhoea.^14^ In the Global Burden of Diseases, Injuries, and Risk Factors study (GBD) 2015, *Cryptosporidium* was highlighted as a leading cause of diarrhoeal mortality in children younger than 5 years old.^15^

We aimed to show whether there is a causal relationship between *Cryptosporidium* and childhood growth and, if so, to quantify the associated additional burden.

## Methods

### Overview

We expanded upon findings from GBD 2016 on the burden of diarrhoeal disease and of undernutrition due to *Cryptosporidium* with a systematic review and meta-analysis on the effect of *Cryptosporidium* on physical growth in children younger than 5 years.

### Estimation of acute burden of diarrhoeal disease due to *Cryptosporidium*

Attribution of *Cryptosporidium* to diarrhoea mortality and morbidity in GBD has been described in detail previously.^15^, ^16^ GBD uses disability-adjusted life-years (DALYs) as a metric of disease burden. DALYs are the sum of years of life lost and years lived with disability.^20^ DALYs due to diarrhoea from *Cryptosporidium* infection, estimated as part of GBD 2016, include diarrhoea episodes and deaths caused by *Cryptosporidium*.^16^ These are referred to from here forward as acute DALYs. The analysis of acute burden uses three separate methodological processes to evaluate the global burden of diarrhoeal diseases; these three processes estimate diarrhoea mortality, diarrhoea morbidity, and diarrhoeal aetiological attribution. Diarrhoea mortality was modelled in a Bayesian ensemble modelling platform that uses space–time trends, data from vital registration systems, and verbal autopsy studies to develop associations with covariates (eg, improved water and sanitation) to predict mortality where model performance is based on in-of-sample and out-of-sample predictive validity.^16^, ^17^ Diarrhoea morbidity was modelled in a Bayesian meta-regression platform that uses a compartmental transition model to relate incidence, prevalence, and mortality.^18^ Sources for this model included population representative surveys, scientific literature, health-care utilisation data, and modelled diarrhoea mortality estimates. Finally, diarrhoeal aetiologies, including *Cryptosporidium* infection, were modelled using a counterfactual approach to estimate a population-attributable fraction (PAF). This method leverages the association between the presence of the aetiology and diarrhoeal symptoms as well as the frequency of detection of the aetiology in diarrhoeal stool samples. The burden due to *Cryptosporidium* was calculated as the product of the *Cryptosporidium* PAF and estimates of diarrhoea morbidity and mortality.

### Estimation of the effect of *Cryptosporidium* on physical growth

We did a systematic review of the scientific literature on the effect of *Cryptosporidium* on physical growth in children younger than 5 years. We searched PubMed on July 26, 2017, with no restriction on publication date, using the search string “cryptospor* AND (stunting[Title/Abstract] OR wasting[Title/Abstract] OR growth[Title/Abstract] OR underweight[Title/Abstract] OR development[Title/Abstract] OR malnutrition[Title/Abstract]) AND Humans[Mesh] NOT (rats or mice)”. We excluded non-human studies. We specifically looked for data describing the change in height or weight either measured in metric units or *Z* scores. Metric units were converted to height-for-age and weight-for-age *Z* scores based on the WHO sex-specific growth curves.^19^

We supplemented the systematic literature review with individual-level data from two case-control and four cohort studies. We did panel-based linear regression models with these data, accounting for an interaction term between *Cryptosporidium* infection and diarrhoea and adjusting for age (days), days between anthropometric measurements, and the previous anthropometric measurement, where measurements for an individual child are treated as repeated measures.

The metadata for the sources can be found in the appendix (p 8). We did a random effects meta-analysis for change in height-for-age *Z* scores (HAZ; stunting), weight-for-age *Z* scores (WAZ; underweight), and weight-for-height *Z* scores (WHZ; wasting) and stratified the results by diagnostic technique used to detect *Cryptosporidium*. These meta-analyses were done in Stata version 13 using the metan function with the random effects option specified. These effect sizes represent the relative change in HAZ, WAZ, and WHZ per episode of diarrhoea from *Cryptosporidium* infection and are used to calculate DALYs attributable to *Cryptosporidium*. We made no distinction in effect size by age.

### Estimation of the burden of undernutrition attributable to *Cryptosporidium*, according to GBD

We also estimated DALYs due to childhood undernutrition in GBD 2016.^21^ The method for calculating undernutrition DALYs has two components. The first is the prevalence of stunting, underweight, and wasting, measured by HAZ, WAZ, and WHZ.^21^ The exposure prevalence of each of these categories are estimated for each country, year, age group, and sex group. The prevalence of underweight and wasting was used in the subsequent analysis of the effect of Cryptosporidium diarrhoea on protein–energy malnutrition.

Undernutrition is a risk factor for diarrhoea, measles, and lower respiratory infections (LRIs) in GBD 2016, based on the statistically significant relative risks from a systematic review.^21^, ^22^ Each of these outcomes has a relative risk given undernutrition status (stunted, underweight, wasted) and the relative risks are adjusted for covariance between undernutrition indicators.^22^

### Estimation of the burden of undernutrition attributable to *Cryptosporidium* infection

To determine the number of undernutrition-associated DALYs attributable to *Cryptosporidium*, we calculated the change, at the population level, in mean HAZ, WAZ, and WHZ due to the pathogen for each age group, year, sex, and geography from GBD. This PAF was defined as:

$$PAF=1-\frac{1}{Diarrhoea\;Episodes\times \mathrm{Pr}oportion_{Crypto}\times \left(\frac{\Delta Zscore}{Crypto\;diarrhoea\;episode}\right)\times RR}$$

Where diarrhoea episodes was the modelled number of diarrhoea episodes (GBD 2016),^18^ Proportion_Crypto_ was the frequency of detection of diarrhoea from *Cryptosporidium* infection (GBD 2016)^16^, Δ*Z* score was the change in *Z* score per episode of diarrhoea from *Cryptosporidium* infection from the meta-analysis, and RR was the relative risk of a given outcome (eg, measles) per *Z*-score change in malnutrition category (ie, per HAZ, WHZ, or WAZ unit change).^22^ We did not calculate an undernutrition PAF for children younger than 1 month because neonatal weight is predominantly related to birthweight.

To calculate a final PAF for undernutrition due to diarrhoea from *Cryptosporidium* infection, we accounted for covariance in WHZ, WAZ, and HAZ. We used the same approach as the risk factor analysis for undernutrition in GBD 2016,^21^ which was defined by:

$$\begin{array}{l} PAF_{undernutrition}=1-[\left(1-PAF_{WAZ}\right)\times \left(1-PAF_{HAZ}\right)\times \\ (1-PAF_{WHZ})] \end{array}$$

The final calculation was to multiply the PAF by the lower respiratory infection, measles, and diarrhoea DALYs estimates to determine the total number of DALYs from those outcomes attributable to *Cryptosporidium* diarrhoea.

### Estimation of the burden of protein–energy malnutrition attributable to *Cryptosporidium* infection

*Cryptosporidium* infection affects weight gain. Protein–energy malnutrition is a burden of disease caused by low weight. To estimate the amount of protein–energy malnutrition that was due to *Cryptosporidium* infection, we estimated the shift in the weight-for-age and weight-for-height distribution due to diarrhoea from *Cryptosporidium* infection. This was done by evaluating the percentage difference in the observed WAZ and WHZ distribution. The shift in the mean WAZ and WHZ at the population level was represented by:

$$\begin{array}{l} Shift=Diarrhoea\;Episodes\;\times \;\mathrm{Pr}oportion_{Crypto}\times \\ \left(\frac{\Delta Zscore}{Crypto\;diarrhoea\;episode}\right) \end{array}$$

And the counterfactual prevalence of wasting and underweight was calculated by:

$$\begin{array}{l} \text{Prevalence}\;\left(Counterfactual\right)=\\ \text{Prevalence}\left(GBD\;2016\;estimate\right)+Shift \end{array}$$

The prevalence of underweight and wasting was converted to a *Z* score, and we estimated the percentage change in the cumulative density from a normal distribution compared with the observed prevalence:

$$PAF\text{=}\frac{\text{Prevalence}\left(GBD\;2016\;estimate\right)-\text{Prevalence}\;\left(Counterfactual\right)}{\text{Prevalence}\left(GBD\;2016\;estimate\right)}$$

PAFs were calculated independently for mild, moderate, and severe undernutrition, age group, geography, sex, and year. Final DALYs due to diarrhoea from *Cryptosporidium* infection were the PAF multiplied by the number of DALYs caused by protein–energy malnutrition.

Uncertainty was carried through the entire analytical process using 1000 draws of the input parameters and modelled estimates. The point values presented are the mean from the 1000 draws, and the lower and upper uncertainty intervals (UIs) are the 2·5th percentile and 97·5th percentile of the 1000 draws.

### Role of the funding source

The funder of the study had no role in study design, data collection, data analysis, data interpretation, or writing of the report. The corresponding author had full access to all the data in the study and had final responsibility for the decision to submit for publication.

## Results

**Figure 1:**
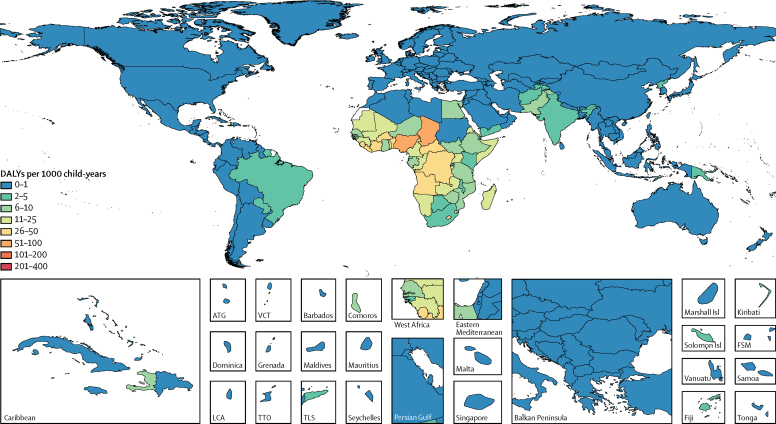
Acute DALYs per 1000 child-years associated with *Cryptosporidium* infection in children younger than 5 years in 2016 DALYs=disability-adjusted life-years. ATG=Antigua and Barbuda. VCT=Saint Vincent and the Grenadines. LCA=Saint Lucia. TTO=Trinidad and Tobago. TLS=Timor-Leste. FSM=Federated States of Micronesia.

In GBD 2016, *Cryptosporidium* was the fifth leading cause of diarrhoeal mortality in children younger than 5 years, causing 48 300 deaths (95% UI 24 600–81 900; table 1). *Cryptosporidium* caused 57 200 deaths (95% UI 29 800–94 700) in all age groups, and more than 80% of deaths from *Cryptosporidium* infection were in children younger than 5 years. 23 300 (48%) of *Cryptosporidium*-related deaths in children younger than 5 years were in Nigeria (n=18 900, 95% UI 9600–33 400) and in the Democratic Republic of the Congo (n=4400, 1700–9000). We estimated 44·8 million episodes of diarrhoea from *Cryptosporidium* infection (95% UI 20·0 million–88·7 million) in children younger than 5 years in 2016, and the incidence was 69·7 per 1000 child-years (95% UI 31·1–137·9; table 1). In 2016, acute diarrhoeal morbidity and mortality from *Cryptosporidium* infection was associated with 4 224 000 DALYs (95% UI 2 160 000–7 163 000) in children younger than 5 years worldwide. The burden of disease varied widely between countries, with the highest burden in the Sahel region of sub-Saharan Africa and in central sub-Saharan Africa (figure 1; table 1). The highest rates of acute *Cryptosporidium* DALYs were in Chad (62·2 per 1000 child-years, 95% UI 16·1–133·1) and Nigeria (55·3 per 1000 child-years, 28·1–97·8; figure 1; appendix p 19). *Cryptosporidium* was not a large cause of diarrhoea burden in high-income countries (571 DALYs, 95% UI 98–1550). The number of acute DALYs due to *Cryptosporidium* infection has decreased significantly between 2006 and 2016 (–42·2%, 95% UI −53% to −28%).

**Table 1:** *Cryptosporidium* deaths, incidence, cases, and DALYs in children younger than 5 years in 2016 in GBD regions

	**Deaths**	**Incidence (per 1000)**	**Episodes**	**Acute DALYs**	**Undernutrition-associated DALYs**	**Total DALYs**	Increase in DALYs*
Global	48 301 (24 612–81 934)	69·7 (31·1–137·9)	44 843 579 (19 989 536–88 698 030)	4 223 986 (2 159 974–7 163 041)	7 851 321 (5 420 602–11 052 124)	12 868 494 (10 148 099–16 010 060)	153%(103–232%)
High-income North America	0 (0–0)	0·2 (0·1–0·3)	4554 (2706–6555)	31 (25–37)	288 (194–410)	319 (226–442)	910%(614–1353%)
Australasia	0 (0–0)	0·2 (0·0–1·0)	272 (76–1769)	6 (1–27)	16 (10–24)	27 (16–51)	180%(45–565%)
High-income Asia Pacific	0 (0–0)	0·1 (0·1–0·2)	805 (439–1359)	6 (4–27)	63 (38–96)	71 (46–105)	863%(398–1596%)
Western Europe	2 (0–6)	7·8 (0·6–27·6)	174 157 (12 306–613 882)	514 (51–1469)	1194 (628–2050)	2429 (1510–4224)	102%(34–237%)
Southern Latin America	0 (0–0)	0·6 (0·3–0·9)	2966 (1620–4477)	14 (10–32)	915 (588–1377)	930 (602–1393)	5891%(3524–9808%)
Eastern Europe	5 (0–12)	21·0 (1·2–62·5)	287 895 (15 793–855 817)	987 (115–2372)	9295 (6454–13 281)	11 102 (7735–16 313)	590%(195–1295%)
Central Europe	1 (0–2)	4·2 (0·8–23·4)	23 994 (4618–133 721)	99 (14–390)	4448 (2861–6513)	4619 (3112–6694)	2797%(1110–5218%)
Central Asia	26 (1–79)	6·3 (0·5–27·6)	68 917 (5206–304 412)	2371 (91–7278)	44 736 (28 312–65 267)	50 277 (32 921–72 541)	982%(252–2434%)
Central Latin America	11 (3–32)	2·3 (0·8–6·1)	52 473 (17 702–136 283)	1064 (293–2969)	23 969 (16 119–33 131)	25 606 (17 623–35 172)	1636%(597–3376%)
Andean Latin America	11 (0–31)	11·6 (0·9–46·9)	76 123 (5975–307 379)	1058 (56–3042)	22 238 (15 572–31 453)	24 589 (17 470–34 027)	1142%(336–2776%)
Caribbean	101 (11–244)	36·6 (1·9–107·5)	151 161 (7802–443 712)	9006 (1043–21 559)	40 278 (21 890–69 605)	54 830 (32 303–89 568)	302%(104–783%)
Tropical Latin America	155 (81–260)	231·6 (103·5–429·1)	3 301 955 (1 475 682–6 117 901)	19 188 (10 637–31 428)	105 931 (79 146–140 758)	126 547 (98 929–161 784)	517%(327–797%)
East Asia	185 (93–334)	17·5 (7·1–38·5)	1 159 312 (470 902–2 544 555)	18 044 (9085–32 364)	32 581 (21 621–47 569)	52 494 (39 330–70 365)	162%(95–289%)
Southeast Asia	164 (9–506)	6·1 (0·6–28·4)	357 786 (36 967–1 669 495)	14 717 (820–46 212)	136 958 (89 043–204 856)	167 615 (113 162–243 030)	517%(150–1300%)
Oceania	17 (5–38)	47·5 (11·6–114·2)	65 568 (16 037–157 718)	1565 (439–3508)	14 043 (6994–26 627)	16 116 (9085–28 598)	689%(259–1644%)
Southern sub-Saharan Africa	719 (294–1295)	103·9 (50·6–193·7)	941 676 (458 361–1 755 910)	63 403 (26 416–113 137)	131 018 (89 442–183 120)	208 445 (159 658–274 770)	170%(97–274%)
Western sub-Saharan Africa	28 396 (13 983–50 422)	252·2 (109·1–492·4)	16 470 528 (7 124 982–32 155 456)	2 463 110 (1 209 737–4 360 397)	3 286 075 (2 123 679–4 930 228)	6 188 684 (4 678 178–7 955 598)	110%(65–188%)
Eastern sub-Saharan Africa	6237 (3079–10 409)	112·9 (52·4–214·4)	7 043 892 (3 268 919–13 374 790)	548 001 (273 263–924 229)	1 493 574 (1 034 601–2 030 080)	2 156 499 (1 689 633–2 743 811)	222%(143–321%)
Central sub-Saharan Africa	7387 (3599–13 036)	439·7 (205·1–841·2)	9 335 506 (4 353 774–17 858 382)	650 614 (320 586–1 139 678)	1 325 341 (777 649–2 119 147)	2 058 400 (1 426 990–2 877 238)	177%(90–336%)

Ranges in parentheses are 95% uncertainty intervals. DALYs=disability-adjusted life-years.

* Relative increase in DALYs when those associated with undernutrition are considered in addition to those associated with diarrhoea (acute DALYs).

Our systematic review retrieved 598 initial results, 49 of which were included for full-text screening, and seven of which provided data (appendix p 8). From our meta-analysis, we calculated that each episode of diarrhoea from *Cryptosporidium* infection significantly decreased HAZ, WAZ, and WHZ (table 2, appendix pp 10–12). When we stratified our analyses by diagnostic method used, the magnitude of the point estimates were only marginally different for PCR-based diagnostics than for non-PCR diagnostic methods (0·06 *vs* 0·04 for HAZ; 0·09 *vs* 0·10 for WAZ), and all meta-analysis data for WHZ used PCR diagnostics. In a meta-analysis of six studies, *Cryptosporidium* infection in the absence of diarrhoea was significantly associated with decreased HAZ (0·030, 95% CI 0·014–0·045; appendix p 13), but infection in the absence of diarrhoea was not significantly associated with WAZ or WHZ. The asymptomatic burden of *Cryptosporidium* was not estimated further.

**Table 2:** Meta-analysis of the effect of *Cryptosporidium* diarrhoea episodes on child growth

	**Mean (95% CI)**	***I*^2^**	**p value**
Height-for-age *Z* score	−0·0469 (−0·0797 to −0·0141)	0·788	0·0051
Weight-for-age *Z* score	−0·0945 (−0·1338 to −0·0055)	0·834	<0·0001
Weight-for-height *Z* score	−0·1256 (−0·1938 to −0·0573)	0·195	0·0031

**Figure 2:**
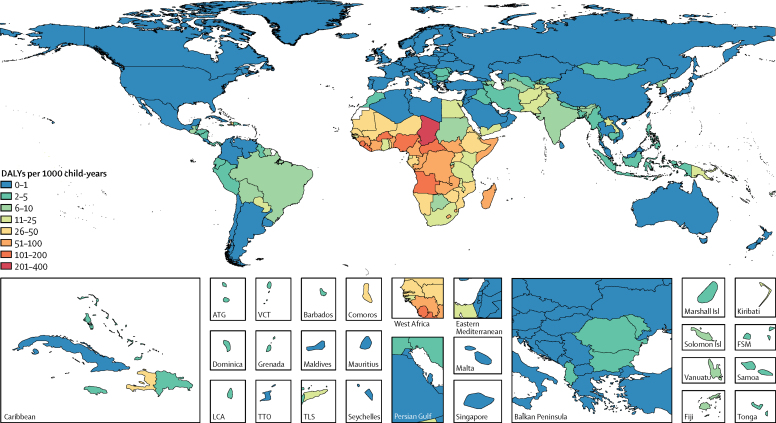
Total (acute and undernutrition-associated) DALYs per 1000 child-years associated with *Cryptosporidium* infection in children younger than 5 years in 2016 DALYs=disability-adjusted life-years. ATG=Antigua and Barbuda. VCT=Saint Vincent and the Grenadines. LCA=Saint Lucia. TTO=Trinidad and Tobago. TLS=Timor-Leste. FSM=Federated States of Micronesia.

**Figure 3:**
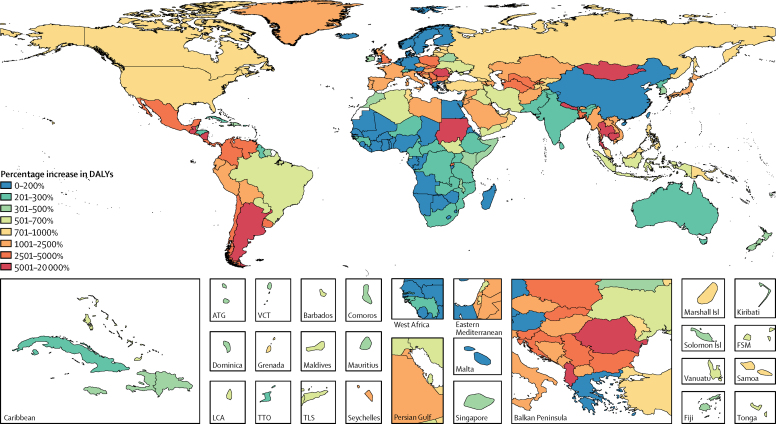
Mean percentage increase in DALYs associated with *Cryptosporidium* infection in 2016 before and after accounting for undernutrition-associated DALYs in children younger than 5 years DALYs=disability-adjusted life-years. ATG=Antigua and Barbuda. VCT=Saint Vincent and the Grenadines. LCA=Saint Lucia. TTO=Trinidad and Tobago. TLS=Timor-Leste. FSM=Federated States of Micronesia.

In GBD 2016, the number of *Cryptosporidium* DALYs affecting children younger than 5 years increased from 4 224 000 DALYs to 12 868 500 DALYs (95% UI 10 148 100–16 010 100) after accounting for undernutrition-associated DALYs—a 153% increase (95% UI 103–232; table 1). The burden of *Cryptosporidium* was highest in sub-Saharan Africa. The highest total rates of DALYs per 1000 child-years occurred in Chad (217·8 per 1000 child-years, 95% UI 132·2–342·1), the Central African Republic (163·7 per 1000 child-years, 108·8–237·8), and Burkina Faso (127·5 per 1000 child-years, 83·1–179·0; figure 2; appendix p 19). These locations also had high rates of acute *Cryptosporidium* DALYs. The countries with the highest percentage increase in *Cryptosporidium* DALYs, after accounting for growth impairment, were Cambodia (18 162% increase, 95% UI 3784–46 818), Guatemala (15 855% increase, 6272–28 108), and Nepal (11 082% increase, 4996 to 20 181; figure 3; appendix p 19). These countries tended to have lower burdens of diarrhoea from *Cryptosporidium* infection but high undernutrition exposure and burdens of lower respiratory infection. These two factors contribute to a large increase in burden due to undernutrition attri-butable to diarrhoea from *Cryptosporidium* infection. The relative number of DALYs increased in every country after accounting for undernutrition-associated DALYs, and although the percentage increase tended to be smallest in high-income, western European countries, DALYs only increased by 70% in Botswana (95% UI 29–161; figure 3; appendix p 19).

**Figure 4:**
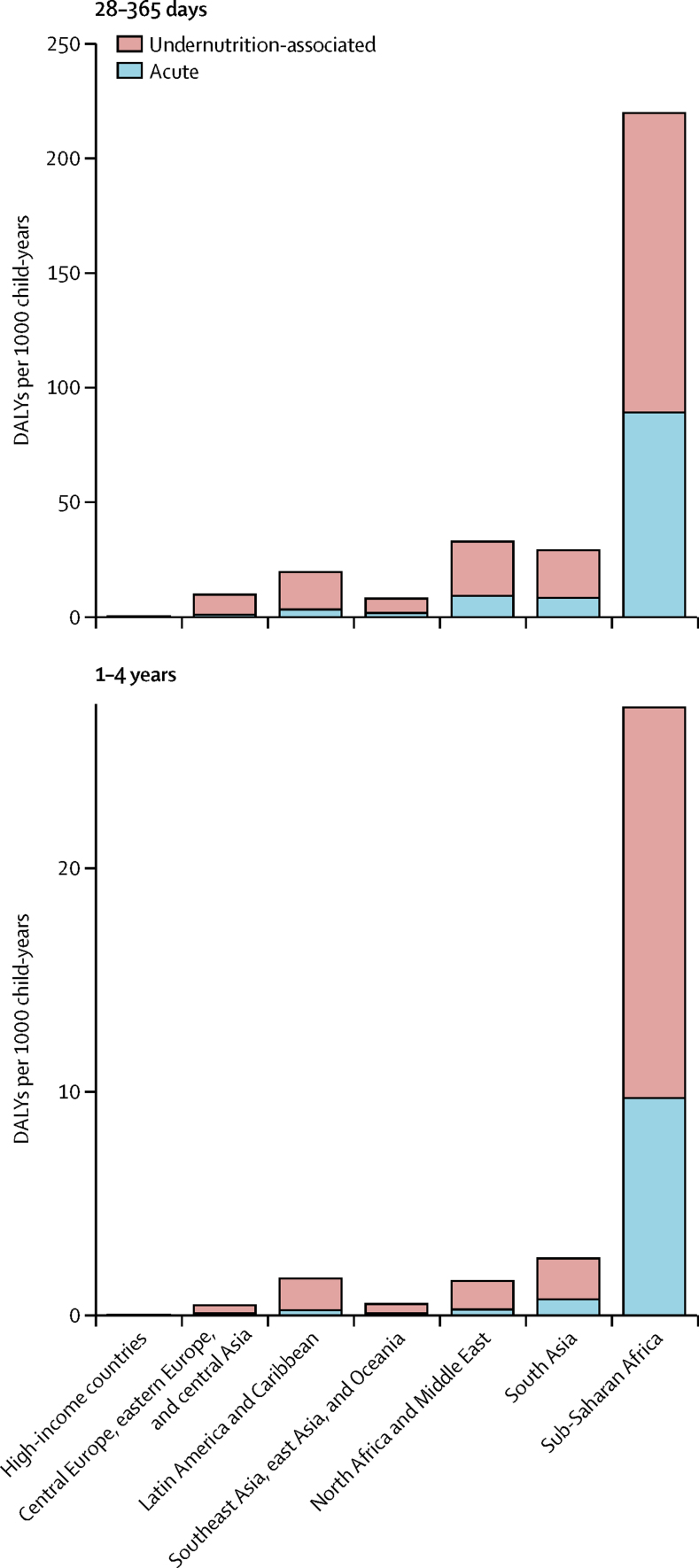
The regional and age distribution of DALYs per 1000 child-years associated with *Cryptosporidium* infection DALYs=disability-adjusted life-years.

**Figure 5:**
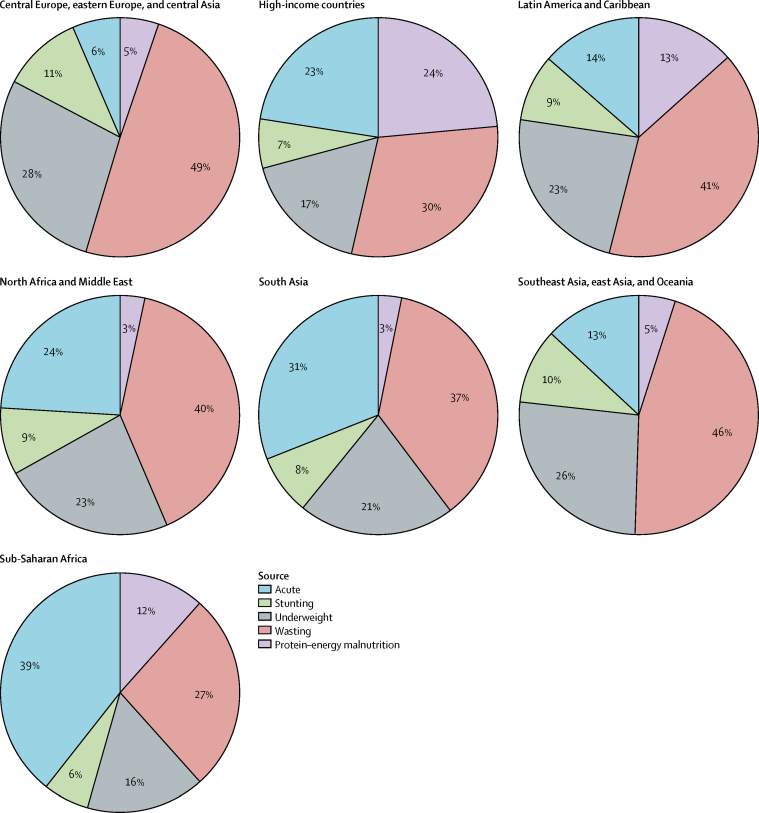
Distribution of the source of DALYs due to *Cryptosporidium* infection in children younger than 5 years in 2016 Distributions are shown for each GBD super-region.

The major burden of *Cryptosporidium* was in children aged 28–364 days (figure 4). After accounting for undernutrition-associated DALYs, *Cryptosporidium* was found to have caused nearly 250 DALYs per 1000 child-years in children younger than 1 year in sub-Saharan Africa, the super-region with the highest burden. Most of this burden is from the long-term outcomes associated with undernutrition. Undernutrition-associated DALYs were associated with 61–94% of all *Cryptosporidium* DALYs (figure 5). Health consequences associated with increased wasting and underweight accounted for most of this additional burden, which exceeded 30% in all super-regions except sub-Saharan Africa (figure 5). The fraction of *Cryptosporidium* DALYs due to protein–energy malnutrition ranged from 3% in north Africa and the Middle East and South Asia to 24% in high-income countries. The acute burden of *Cryptosporidium* ranged from 6% in central Europe, eastern Europe, and central Asia to 39% in sub-Saharan Africa (figure 5).

## Discussion

Our findings show that the previously reported burden of *Cryptosporidium* was an underestimation of the true burden. Diarrhoea from *Cryptosporidium* infection affects childhood health beyond acute illness by decreasing growth, particularly weight gain, and increasing the risk of subsequent infectious disease episodes. By properly accounting for some of these long-term outcomes, we estimated that the burden of *Cryptosporidium* is 2·5 times higher than previously reported. Our findings call for renewed efforts to control the burden of *Cryptosporidium.*

*Cryptosporidium* was the fifth leading cause of diarrhoeal mortality in children younger than 5 years in 2016.^16^ The importance of *Cryptosporidium* as a cause of moderate-to-severe diarrhoea has been identified elsewhere.^14^ There are also geographic patterns to the burden of *Cryptosporidium*; the burden appears to be focused in central and western sub-Saharan Africa, which is an area with a high risk of infectious disease. This is also an area of the world with relatively sparse data on the burden of diarrhoea and *Cryptosporidium* aetiology, which should be recognised in the interpretation of our estimates.

There are several reasons for suspecting that diarrhoea and *Cryptosporidium* infection impair physical growth.^23^, ^24^ The rapid fluid loss and inability to absorb macronutrients and micronutrients during a diarrhoeal episode disrupts weight and height gain. Furthermore, chronic or repeated enteric infections are thought to disrupt normal gut function by changing endothelial cells, causing chronic inflammation, and flattening the microvilli, which will decrease the area of absorptive tissue.^25^, ^26^, ^27^ Invasive, intracellular infections such as those caused by *Cryptosporidium* might be more difficult to clear, causing long-lasting inflammation in the enteric system and disruption of the epithelial cell barrier.^4^, ^28^ Low weight, particularly weight-for-height, is serious risk factor for infectious diseases.^22^ Low weight has immediate, acute negative effects on systematic and mucosal immune system functions.^23^, ^29^ Poor height gain is a long-term negative outcome, and if height growth is not restored by the end of the first 2 years of life, it becomes more difficult to recover later in childhood development. Evidence suggests that the failure to meet genetic growth potential could induce poor health outcomes that are lifelong and possibly intergenerational, including cognitive development, poor educational performance, and increased risk of cardiovascular and metabolic diseases in later life.^30^, ^31^, ^32^

These data on the distribution of disease burden attributable to *Cryptosporidium* will allow policy makers and research funders to prioritise programmes and interventions that maximise health and long-term outcomes. Additionally, our work draws attention to important knowledge gaps and research priorities. Considerable uncertainty about the nature of the *Cryptosporidium* burden remains. Even less is known about the long-term consequences of infection, such as the link between cognitive development and the stunting due to *Cryptosporidium* infection.^33^ We did not distinguish between acute, persistent, or prolonged diarrhoea, yet evidence suggests that *Cryptosporidium* is associated with an extended duration of diarrhoea and that long-lasting diarrhoea could affect the long-term burden non-linearly.^34^ Our findings call for further investigations about the prevalence of asymptomatic infection and how such infections can also lead to morbidity. Although our data suggest that *Cryptosporidium* infection in the absence of diarrhoea might impede linear growth, quantifying the prevalence of asymptomatic infection was beyond the scope of this work. Attention to these limitations could have important implications for which interventions will best address the *Cryptosporidium* burden through prevention and treatment.

In terms of scalable interventions, no vaccine for *Cryptosporidium* exists, and unfortunately, in view of scientific, logistical, and economic challenges, the development timeline is expected to be long.^35^ Effective antiparasitic vaccines to prevent infection of human beings and disease have historically been quite challenging to develop.^36^ Improved knowledge about how *Cryptosporidium* behaves within other organisms, increased understanding of the host's immune response to infection by the parasite, and the annotation of *Cryptosporidium* genomes essential to fill crucial gaps in our knowledge of how the pathogen behaves. Despite substantial efforts, no antiparasitic vaccine for use in human beings has been licensed to date. For example, the antiparasitic vaccine closest to approval for human use, the RTS,S malaria vaccine candidate, has been in development for more than 30 years.^36^ Furthermore, available therapeutic options for cryptosporidiosis hardly exist for most wild-type infections in children living in developing countries. These children, particularly those with immunocompromising conditions such as HIV, are at highest risk of severe disease and long-term sequelae.^37^ In 2002, the US Food and Drug Administration approved nitazoxanide for the treatment of *Cryptosporidium* and *Giardia duodenalis* infections in children aged 1–11 years. Nitazoxanide might be 38% effective at treating protozoal diarrhoeal infections in immunocompetent people, but it does not appear to be as efficacious in these high-risk populations.^38^, ^39^

In addition to managing dehydration with fluids and oral rehydration solutions, effective therapeutic strategies for *Cryptosporidium* infection might depend on accurate point-of-care diagnostics. Existing diagnostic tests include microscopy of stool for oocysts, immunofluorescent assay, dipstick ELISA, and PCR, but a rapid, low-tech, sensitive, specific, and affordable diagnostic test that is suitable for low-resource settings is not yet available. Studies have shown that quantitative PCR is more sensitive for detecting *Cryptosporidium* than traditional laboratory methods,^40^ so non-molecular diagnostics might underestimate the true prevalence. The sensitive diagnostic technologies have detected *Cryptosporidium* in a sizeable percentage (8·5%) of moderate-to-severe diarrhoea cases. In most of these cases, *Cryptosporidium* was considered the probable causative or dominant pathogen.^41^

There are several important limitations to this study. Broadly, data from high-burden areas of the world are scarce. Our study required statistical prediction models that are based on a variety of input data types such as mortality, diarrhoea incidence, *Cryptosporidium* detection frequency, prevalence of childhood undernutrition, and other infectious disease burden. These models predict estimates even where there are no data, relying on space–time and covariate information. However, every step in our estimation process is documented, input data are publicly available, and uncertainty is propagated through the entire process, per the Guidelines for Accurate and Transparent health Estimates Reporting (GATHER).^42^ Children that have poor growth could be at increased risk of *Cryptosporidium* infection, and this cyclical association introduces a challenge in isolating the temporal relationship between diarrhoea from *Cryptosporidium* infection and physical growth. However, where possible, data for this study are of growth after diarrhoea from *Cryptosporidium* infection. Relatively few data are available for the analysis to quantify the relation between diarrhoea from *Cryptosporidium* infection and childhood growth indicators. We found a difference in the effect sizes when stratifying by the diagnostic method used and these findings suggest that quantitative molecular diagnostics may improve our understanding of *Cryptosporidium* diarrhea on childhood growth. Our results might also depend on the age of infection, suggesting that the effect of diarrhoea from *Cryptosporidium* infection is higher in children under 1 year than in older children. One further bias might arise from the consistency in the input data. We found some variability in the sources used in the meta-analyses; the I^2^ values from the meta-analyses suggested wide dispersion in the data, a limitation that should be partly accounted for in our use of a random-effects meta-analysis.

We did not examine economic burden and societal costs for *Cryptosporidium* infection, which is suggested to be substantial and to far outweigh direct treatment costs.^43^, ^44^ Rafferty and colleagues^45^ examined direct and indirect costs associated with symptomatic cryptosporidiosis in infants younger than 12 months in Kenya, Peru, and Bangladesh and estimated that direct costs per *Cryptosporidium* infection were highest in Kenya (US$59·01), Peru ($23·32), and Bangladesh ($7·62).

Our work highlights the acute and long-term effects of infection in children younger than 5 years and illustrates the unequitable distribution of that burden in low-income geographies. Interventions to improve childhood health and wellbeing, such as through improved water and sanitation sources and breastfeeding promotion, and a renewed urgency in the development of *Cryptosporidium* prevention and treatment options could ensure that all children have an opportunity to grow, thrive, and live healthy lives.
