# Supplementary material for: Morbidity, mortality, and long-term consequences associated with diarrhoea from Cryptosporidium infection in children younger than 5 years: a meta-analyses study
Source: Lancet Glob Health. 2018 Jun 13;6(7):e758–68. doi: 10.1016/S2214-109X(18)30283-3 (PMC6005120; doi:10.1016/S2214-109X(18)30283-3)
Supplement: Supplementary appendix [file mmc1.pdf]

THE LANCET
Global Health

Supplementary appendix

This appendix formed part of the original submission and has been peer reviewed. We post it as supplied by the authors.

Supplement to: Khalil IA, Troeger C, Rao PC, et al. Morbidity, mortality, and long-term consequences associated with diarrhoea from *Cryptosporidium* infection in children younger than 5 years: a meta-analyses study. *Lancet Glob Health* 2018; **6:** e758–68.

Supplementary Material: Morbidity, mortality, and long-term consequences associated with diarrhoea from *Cryptosporidium* infection in children younger than 5 years: a meta-analyses study

Supplementary Tables

Supplementary Table 1. Data points used for Cryptosporidium modelling. 4
Supplementary Table 2. Sources included in the Cryptosporidium effect size meta-analyses. 8
Supplementary Table 3. Cryptosporidium deaths, incidence, cases, DALYs among children under 5 in 2016 by country 19

Supplementary Figures

Supplementary Figure 1. Number of unique data sources for Cryptosporidium proportion modelling by country. 3
Supplementary Figure 2. Relationship between qPCR cycle threshold and diagnostic accuracy 6
Supplementary Figure 3. Forest plot of the effect per Cryptosporidium diarrhoea episode on height-for-age z-scores based on a random effects meta-analysis 10
Supplementary Figure 4. Forest plot of the effect per Cryptosporidium diarrhoea episode on weight-for-age z-scores based on a random effects meta-analysis 11
Supplementary Figure 5. Forest plot of the effect per Cryptosporidium diarrhoea episode on weight-for-height z-scores based on a random effects meta-analysis 12
Supplementary Figure 6. The effect of non-diarrhoea Cryptosporidium infection on height-for-age z-scores from a random effects meta-analysis 13
Supplementary Figure 7. Sensitivity analyses of the effect of Cryptosporidim diarrhoea on height-for-age and weight-for-age z-scores. 14

Supplementary Material

This document provides supplementary material for the manuscript ***Morbidity, mortality, and long-term consequences associated with diarrhoea from* Cryptosporidium *infection in children younger than 5 years: a meta-analytic study.*** The information in the document is intended to provide additional detail for data extraction and modelling strategy.

Overview

The burden of *Cryptosporidium* is modelled in two ways in this study. The first is the acute burden due to incident episodes and deaths due to *Cryptosporidium* diarrhoea and is the primary output of the Global Burden of Disease study. The second is quantifying the impact of *Cryptosporidium* diarrhoea on childhood physical growth.

Diarrhoea mortality and morbidity in GBD 2016

The Global Burden of Disease study 2016 provides comprehensive and internally consistent epidemiological estimates for over 300 causes of death and disability, 90 risk factors, for 195 locations, by year, sex, and four age groups under 5 years old. Detailed descriptions of all GBD methods have been previously published.[1–4] Mortality due to diarrhoeal diseases was estimated using the Cause of Death Ensemble modeling framework (CODEm)[2,5] and diarrhoea incidence, prevalence, and recovery were estimated with DisMod-MR 2.1 (DisMod), a Bayesian meta-regression tool.[6] More detail on diarrhoea modeling in GBD can be found several places including the GBD 2015 diarrhoea capstone manuscript,[1] the GBD 2016 cause of death publication[2] methods appendix (p. 58) and the GBD 2016 non-fatal publication[3] methods appendix (p. 54). The diarrhoea incidence, mortality, years of life lost (YLLs), years lived with disability (YLDs), and disability-adjusted life years (DALYs) estimated in the GBD 2016 framework are referred to herein as acute DALYs to differentiate them from DALYs associated with growth impairment which will be referred to as long-term sequelae DALYs. DALYs associated with growth impairment will be described in more detail below; we estimated these DALYs as part of the GBD 2016 and estimated these DALYs attributable to diarrhoea in children under-5.

Cryptosporidium burden in GBD 2016

The modelling strategy for *Cryptosporidium* diarrhoea in GBD has been described elsewhere.[1] Etiologic attribution is estimated separately from diarrhoea mortality and morbidity. Diarrhoeal etiologies were attributed using a counter-factual approach called a population attributable fraction (PAF).[1,2] Our approach accounted for pathogen co-detection and detection in healthy individuals, and does not necessitate a one pathogen to one episode relationship. The population attributable fraction is defined as:[7]

$$PAF = Proportion * (1-1/OR)$$

The odds ratios represent the relationship between the detection of *Cryptosporidium* and moderate-to-severe diarrhoea as quantified using molecular diagnostics from the Global Enteric Multicenter Study.[8,9] There are two odds ratios, one for children under-1 (3.09, 95% Confidence Interval [CI] 2.21-4.28) and one for all older age groups (1.83, 95% CI 1.3-2.44). The odds ratios were estimated in a conditional logistic mixed-effects regression model that included all other pathogens in the model with an interaction between *Cryptosporidium* and age.

The Proportion is the frequency of detection, where the molecular diagnostic is the case definition, of *Cryptosporidium* in diarrhoeal stool samples and is a modelled estimate for each age, year, sex, and geography. The number of data points by each country is shown in the map below and summarised in the table below. A data point represents the most specific estimate of the proportion of diarrhoea where *Cryptosporidium* was present by age-sex-year. Data were extracted and modeled by age-sex-location-year. For this reason, an individual study must contribute at least one data point but can contribute many to the modeling of *Cryptosporidium* proportion.

Supplementary Figure 1. Number of unique data sources for *Cryptosporidium* proportion modelling by country.

Data sources were identified from the scientific literature.

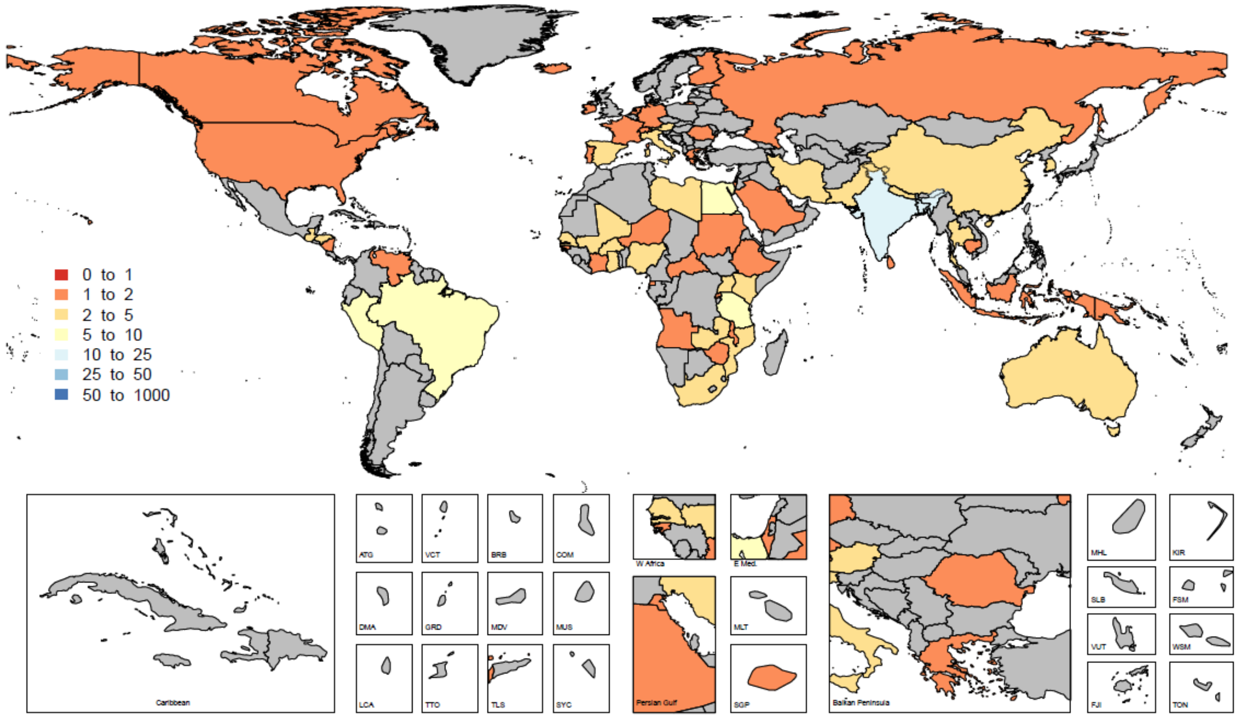

Supplementary Table 1. Data points used for *Cryptosporidium* modelling.

Data points represent the number of unique values for the proportion of diarrhoea episodes where *Cryptosporidium* was detected. The number of data points used in *Cryptosporidium* proportion modelling by geography is shown. The number from an inpatient population and the number of data points that tested for *Cryptosporidium*, excluding other diarrhoeal etiologies, are shown in the second and third columns.

Geography	Data points	From inpatient sample population	*Cryptosporidium* tested only
Angola	2	1	0
Australia	5	2	1
Austria	3	0	0
Bangladesh	32	17	5
Brazil	12	5	0
Burkina Faso	9	0	1
Cambodia	1	0	0
Canada	8	0	0
Central African Republic	3	3	0
China	11	0	0
Cote d'Ivoire	1	0	0
Egypt	23	5	9
England	2	0	0
Ethiopia	10	10	10
Finland	2	0	0
France	2	0	0
Germany	2	0	0
Ghana	3	0	1
Greece	2	0	0
Guatemala	11	1	0
Guinea-Bissau	5	0	0
Honduras	2	0	0
Iceland	5	0	0
India	66	46	4
Indonesia	16	8	8
Iran	20	16	8
Ireland	2	0	0
Israel	1	1	0
Italy	13	10	4
Kenya	18	9	9
Kuwait	5	0	0
Libya	5	4	0
Malawi	1	0	1
Mali	15	9	0
Mozambique	18	9	0

Nepal	5	0	0
Netherlands	5	5	0
Nicaragua	3	0	0
Niger	1	0	1
Nigeria	9	0	0
Northern Ireland	2	0	0
Pakistan	18	9	0
Papua New Guinea	5	5	0
Peru	12	5	0
Portugal	1	0	0
Romania	1	0	0
Russia	1	0	0
Rwanda	1	1	0
Sao Tome and Principe	1	1	0
Saudi Arabia	2	2	0
Scotland	2	0	0
Senegal	3	1	0
Singapore	1	0	0
South Africa	11	1	0
South Korea	24	4	20
Spain	13	0	0
Sri Lanka	1	0	0
Sudan	8	0	0
Switzerland	1	1	0
Tanzania	14	5	0
Thailand	2	1	0
The Gambia	16	9	1
Uganda	4	4	3
United States	6	1	0
Venezuela	1	0	0
Wales	2	0	0
Western Europe	3	0	0
Zambia	6	6	0
Zimbabwe	6	0	6

Many studies investigating the etiology of diarrhoea tested for the presence of *Cryptosporidium* using ELISA or microscopy. In order to make these estimates comparable with our case definition from quantitative polymerase chain reaction (qPCR), we had to make several data adjustments. The first was to determine a cut point in the continuous qPCR test result that differentiates between negative and positive results. To do this, we found the cycle threshold (Ct) that maximised the diagnostic accuracy in discriminating between cases and controls in GEMS. Functionally this represents the point that is most likely to dichotomise test results between *Cryptosporidium* infection likely to cause diarrhoea and *Cryptosporidium* infection unlikely to cause diarrhoea or no

Cryptosporidium. A Ct value of 35 indicates that no *Cryptosporidium* gene target was identified in a sample. This is equivalent to determining the Youden's index- maximizing sensitivity and specificity. The relationship between diagnostic accuracy and Ct is shown below.

Supplementary Figure 2. Relationship between qPCR cycle threshold and diagnostic accuracy

This figure shows the relationship between the qPCR cycle threshold and the accuracy in discriminating between cases and controls. The blue curve indicates a loess curve fitted to the data while the vertical blue line indicates the cut-point determined in this analysis. Any Ct value below the blue line indicates a positive test result for *Cryptosporidium.*

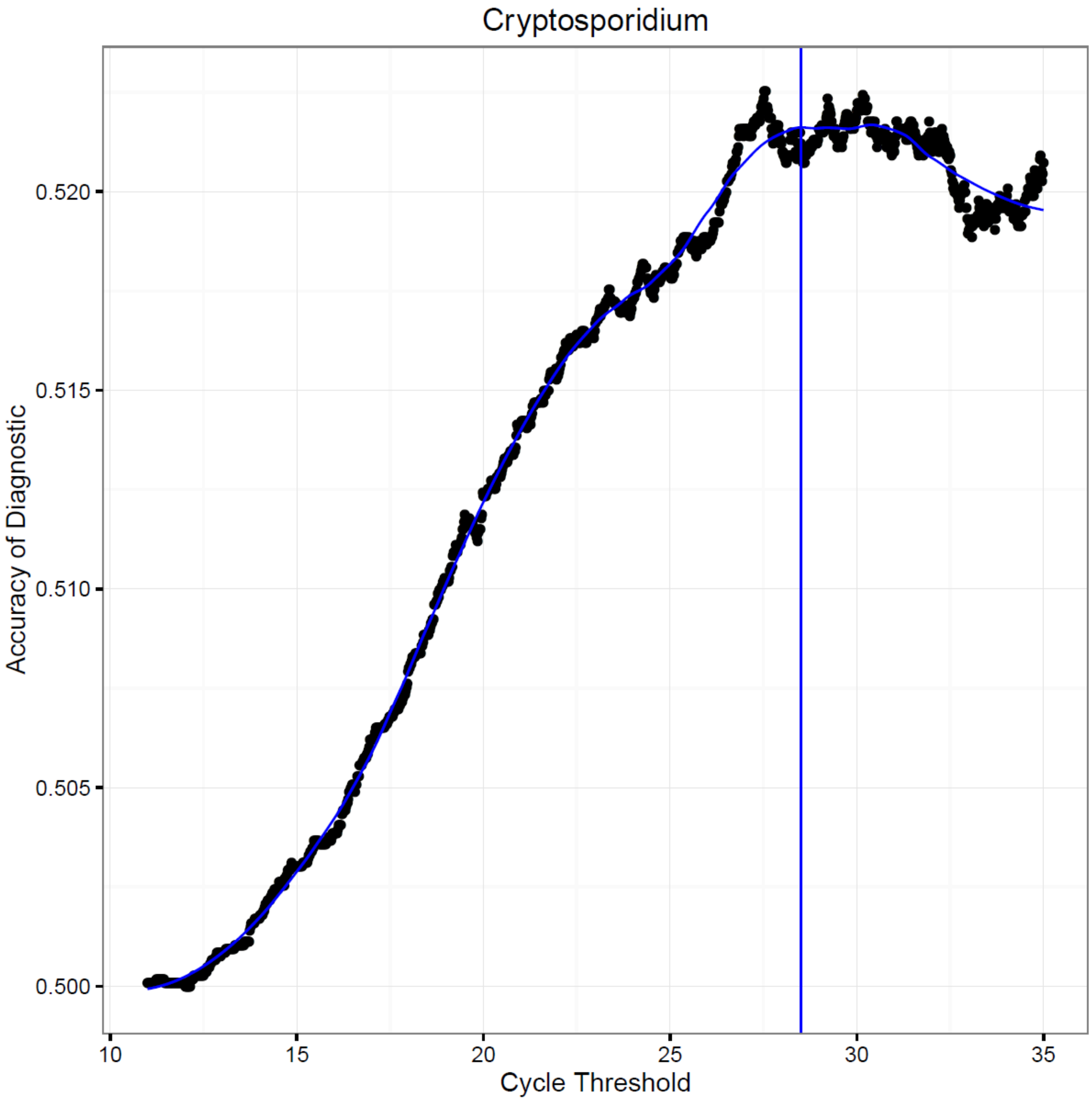

Growth faltering due to *Cryptosporidium*

Our primary long-term outcomes of interest were changes in Z-scores of height or length for age (HAZ), weight for age (WAZ), and weight for height (WHZ) subsequent to diarrhoea. We defined mild, moderate, and severe stunting, underweight, and wasting as -1 to -2, -2 to -3, and <-3 Z-scores of HAZ, WAZ, and WHZ, respectively. When possible, HAZ, WAZ, and WHZ were defined according to the World Health Organization (WHO) 2006 growth charts[10], rather than the CDC 2000[11] or the 1977 National Center for Health Statistics (NCHS) growth charts. We selected these outcomes because they are health related, quantifiable, have sufficient literature or available microdata for meta-analyses, and are available in the Global Burden of Disease Study (GBD).[1,4]

We performed a systematic review of published scientific literature for the impact of *Cryptosporidium* on physical growth in children under five years old. The PubMed search was performed in July 2015 and updated in July 2017. The search string is provided below and returned 598 results.

> *cryptospor* AND (stunting[Title/Abstract] OR wasting[Title/Abstract] OR growth[Title/Abstract] OR underweight[Title/Abstract] OR development[Title/Abstract] OR malnutrition[Title/Abstract]) AND Humans[Mesh] NOT (rats or mice)*

From these 598 initial results, 49 were included for full-text screening and 7 provided data. We specifically looked for data describing the change in height or weight either measured in metric units or z-scores. Metric units were converted to height-for-age and weight-for-age z-scores based on the WHO sex-specific growth curves.

We supplemented the systematic literature review with individual-level data from several case control and cohort studies. We performed panel-based linear regression models with these data accounting for an interaction term between *Cryptosporidium* and diarrhoea, and adjusting for age in days, days between anthropometric measurements, and the previous anthropometric measurement.

The input data can be found in Table S2.

Supplementary Table 2. Sources included in the *Cryptosporidium* effect size meta-analyses.

Gray sources are ones for which individual-level microdata were used.

First Author or Primary Investigator	Location	Citation	Input data
Agnew, DG[12]	Fortaleza, Brazil	Agnew DG, Lima AA, Newman RD, *et al.* Cryptosporidiosis in northeastern Brazilian children: association with increased diarrhea morbidity. *J Infect Dis* 1998; **177**: 754–60.	HAZ
Checkley, W[13]	Lima, Peru	Checkley W, Epstein LD, Gilman RH, Cabrera L, Black RE. Effects of Acute Diarrhea on Linear Growth in Peruvian Children. *Am J Epidemiol* 2003; **157**: 166–75.	Height (cm)
Checkley, W[14]	Lima, Peru	Checkley W, Gilman RH, Epstein LD, *et al.* Asymptomatic and symptomatic cryptosporidiosis: their acute effect on weight gain in Peruvian children. *Am J Epidemiol* 1997; **145**: 156–63.	Weight (kg)
Kang, C[15]	Vellore, India	India - Vellore Birth Cohort *Cryptosporidium* Immune Response Study 2009-2013	HAZ, WAZ, WHZ
Korpe, PS[16]	Bangladesh	Korpe PS, Haque R, Gilchrist C, Valencia C, Niu F, Lu M, Ma JZ, Petri SE, Reichman D, Kabir M, Duggal P, Petri WA. Natural History of Cryptosporidiosis in a Longitudinal Study of Slum-Dwelling Bangladeshi Children: Association with Severe Malnutrition. *PLoS Negl Trop Dis*. 2016; 10(5): e0004564.	HAZ
Kotloff, K[17]	Multiple	GEMS-1A: Center for Vaccine Development, University of Maryland, Centers for Disease Control and Prevention (CDC), Department of Medical Microbiology and Immunology, Göteborg University, International Centre for Diarrhoeal Disease Research, Bangladesh (ICDDR,B), International Vaccine Institute, Perry Point Cooperative Studies Program Coordinating Center, US Department of Veterans Affairs, School of Medicine, University of Virginia, University of Chile. Global Enteric Multicenter Study 2011-2013.	HAZ
Kotloff, K[8]	Multiple	Kotloff KL, Nataro JP, Blackwelder WC, Nasrin D, Farag TH, Panchalingam S, Wu Y, Sow SO, Sur D, Breiman RF, Faruque AS, Zaidi AK, Saha D, Alonso PL, Tamboura B, Sanogo D, Onwuchekwa U, Manna B, Ramamurthy T, Kanungo S, Ochieng JB, Omore R, Oundo JO, Hossain A, Das SK, Ahmed S, Qureshi S, Quadri F, Adegbola RA, Antonio M, Hossain MJ, Akinsola A, Mandomando I, Nhampossa T, Acácio S, Biswas K, O'Reilly CE, Mintz ED, Berkeley LY, Muhsen K, Sommerfelt H, Robins-Browne RM, Levine MM. Burden and aetiology of diarrhoeal disease in infants and young children in developing countries (the Global Enteric Multicenter Study, GEMS): a prospective, case-control study. *Lancet*. 2013; 382(9888): 209–22.	HAZ
Lima, AA[18]	Fortaleza, Brazil	Lima AA, Moore SR, Barboza MS, *et al.* Persistent diarrhea signals a critical period of increased diarrhea burdens and nutritional shortfalls: a prospective cohort study among children in northeastern Brazil. *J Infect Dis* 2000; **181**: 1643–51.	HAZ, WAZ
MAL-ED Network Investigators[19]	Multiple	MAL-ED Network Investigators. Etiology, Risk Factors, and Interactions of Enteric Infections and Malnutrition and the Consequences for Child Health (MAL-ED) Study.	HAZ, WAZ, WHZ
Molbak, OK[20]	Bandim, Guinea-Bissau	Mølbak K, Andersen M, Aaby P, *et al.* *Cryptosporidium* infection in infancy as a cause of malnutrition: a community study from Guinea-Bissau, west Africa. *Am J Clin Nutr* 1997; **65**: 149–52.	Height (cm), weight (kg)
Moore, SR[21]	Fortaleza, Brazil	Moore SR, Lima NL, Soares AM, *et al.* Prolonged episodes of acute diarrhea reduce growth and increase risk of persistent diarrhea in children. *Gastroenterology* 2010; **139**: 1156–64.	HAZ, WAZ
Petri, W[22]	Dhaka, Bangladesh	Center for Public Health Genomics, Centers for Disease Control and Prevention (CDC), International Vaccine Institute, School of Medicine, University of Vermont, University of Virginia. Bangladesh - Dhaka Performance of Rotovirus and Oral Vaccines in Developing Countries Study 2011-2014	HAZ, WAZ, WHZ
Petri, W	Dhaka, Bangladesh	International Centre for Diarrhoeal Disease Research, Bangladesh (ICDDR,B). Bangladesh - Dhaka Cohort Study of Cryptosporidiosis in Children 2014. [Unpublished]	HAZ, WAZ, WHZ

To standardise the change in growth associated with *Cryptosporidium* diarrhoea, we use height-for-age z-scores and weight-for-age z-scores. The HAZ and WAZ values are intended to be universally usable and are produced by the World Health Organization. Some studies report changes in growth as change in HAZ or WAZ. For studies that don't, we convert the change in weight (kilograms) and in height (centimeters) to z-scores using the WHO Growth Charts (http://www.who.int/childgrowth/standards/en/). At a given age, the difference in weight or height per z-score is used to convert to z-scores for analysis. When possible, we use sex specific z-scores but if a study makes no distinction between sexes, we use the z-score for girls. The age for the z-score lookup is the median age in the time period in months.

The WAZ distribution is assumed to be right-tailed, meaning that the standard deviations below the median get progressively smaller and those above the median get progressively larger. For this analysis, the kilograms/z-score change are the first standard deviation below the median.

A number of studies report p-values on associations but fail to report the confidence intervals. To approximate the confidence intervals, we use the equation [23]:

$$Z = -0.862 + \sqrt{(0.743 - 2.404 * \ln(p))}$$

$$Standard\ Error = \frac{Estimate}{Z}$$

$$CI = Estimate - SE * 1.96; Estimate + SE * 1.96$$

We performed a series of random effects meta-analyses to summarise the relationship between childhood growth indicators, HAZ, WAZ, and WHZ, and *Cryptosporidium* diarrhoea. We quantified the effect in z-scores on each of these indicators per episode of *Cryptosporidium* diarrhoea. The results of these analyses are shown in Figures S3-S5. We also performed a random effects meta-analysis on the impact of *Cryptosporidium* infection in the absence of diarrhoea on each indicators. This relationship was statistically significant only for change in HAZ, shown in Figure S6.

Supplementary Figure 3. Forest plot of the effect per *Cryptosporidium* diarrhoea episode on height-for-age z-scores based on a random effects meta-analysis

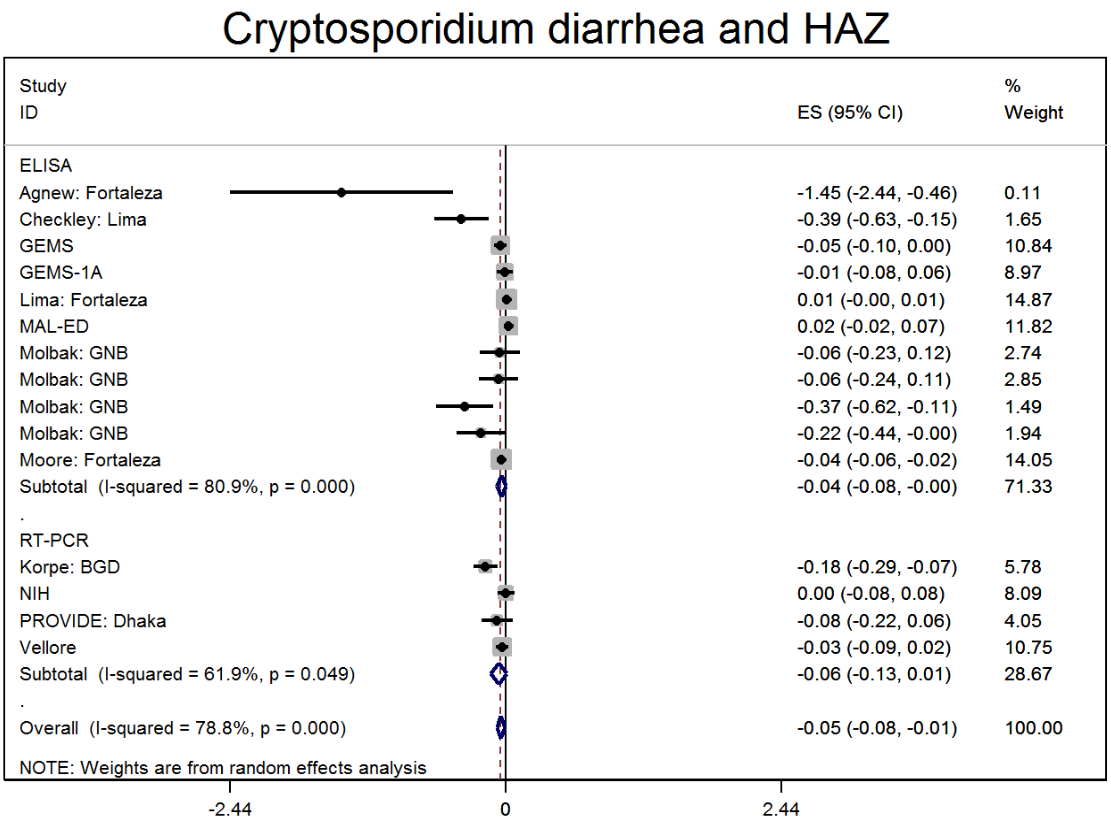

Supplementary Figure 4. Forest plot of the effect per *Cryptosporidium* diarrhoea episode on weight-for-age z-scores based on a random effects meta-analysis

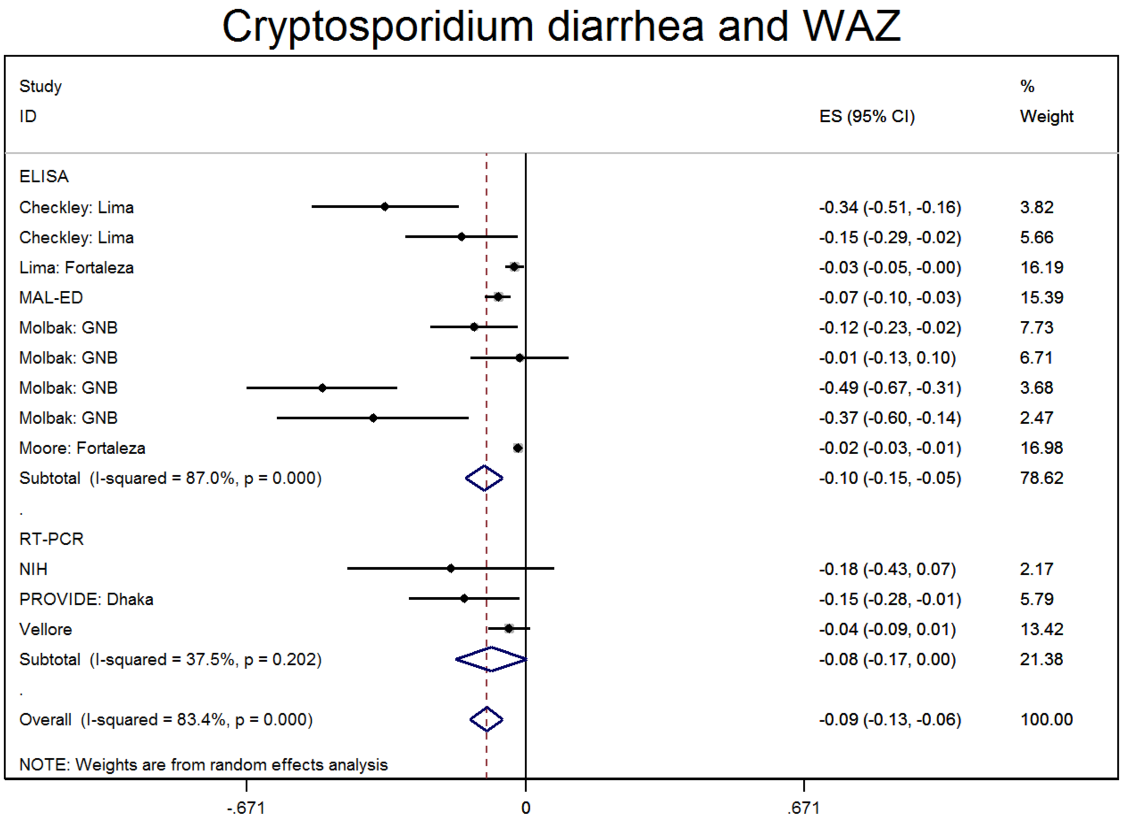

Supplementary Figure 5. Forest plot of the effect per *Cryptosporidium* diarrhoea episode on weight-for-height z-scores based on a random effects meta-analysis

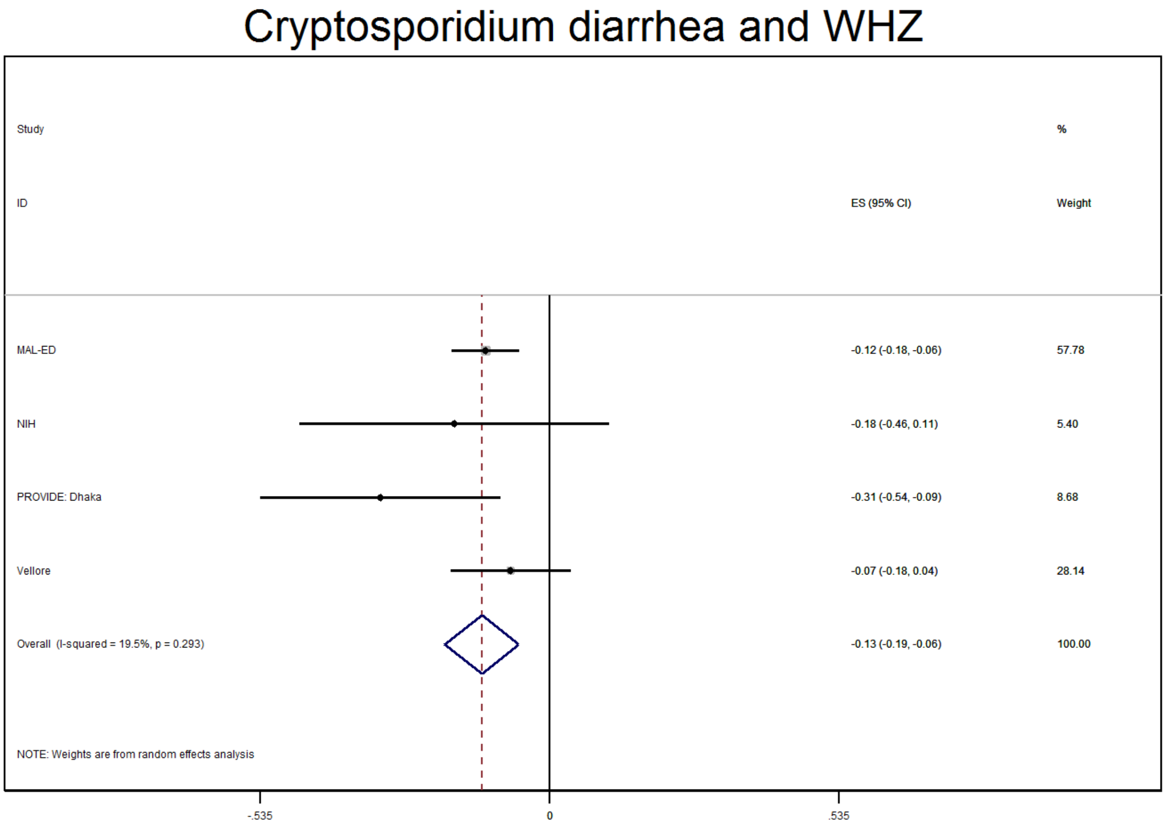

Supplementary Figure 6. The effect of non-diarrhoea *Cryptosporidium* infection on height-for-age z-scores from a random effects meta-analysis

The point estimates in the forest plot indicate the summary estimate of the change in HAZ scores wherein *Cryptosporidium* infection in the absence of diarrhoea was identified between anthropometric measurements.

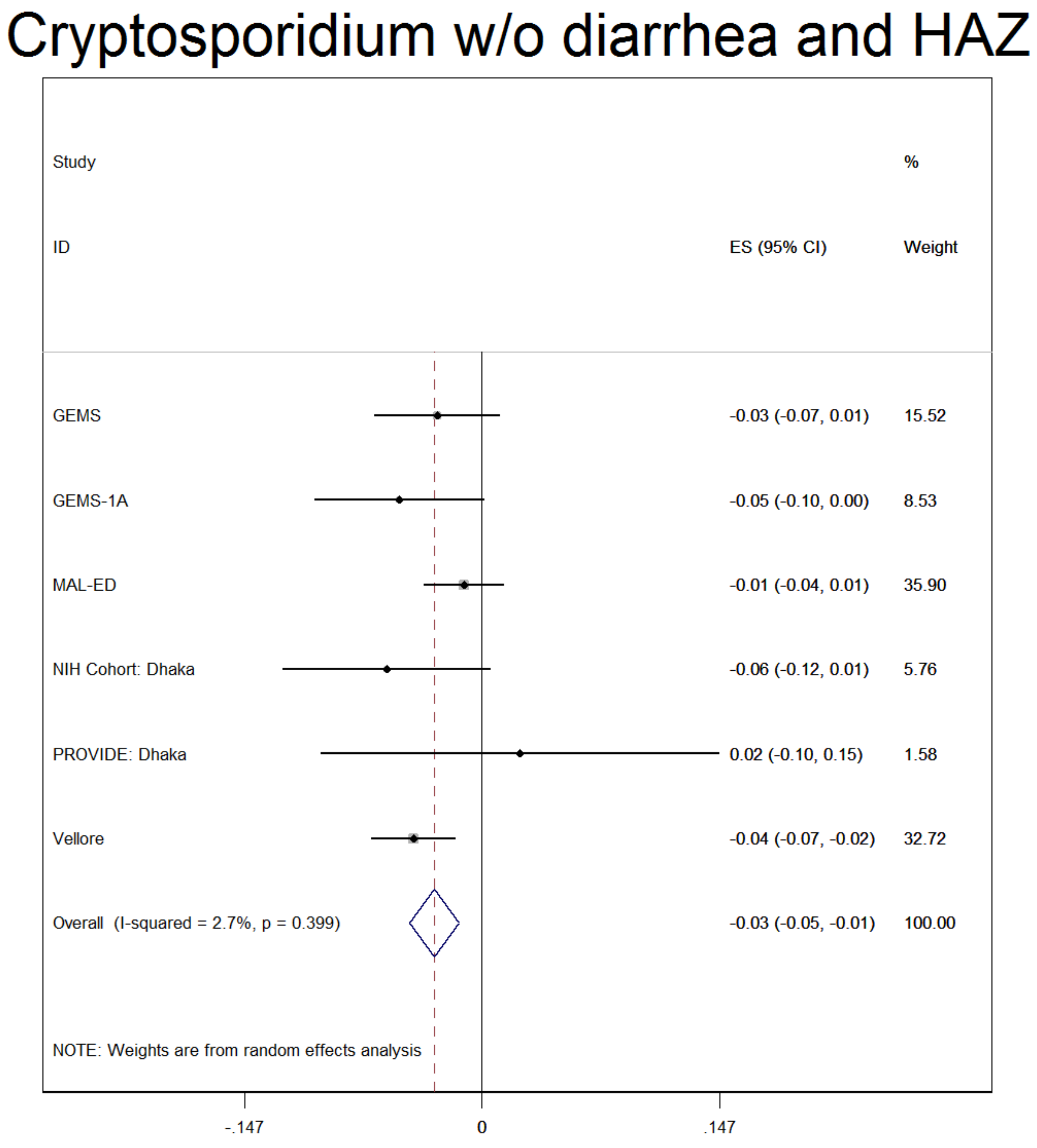

Supplementary Figure 7. Sensitivity analyses of the effect of Cryptosporidim diarrhoea on height-for-age and weight-for-age z-scores.

The analysis is stratified by age of exposure (A and B) and by data source type (C and D). There were insufficient data to perform these analyses for weight-for-height. Note that the results in these forest plots are not exactly the same as the primary analyses because the input data were stratified for these sensitivity analyses.

A)

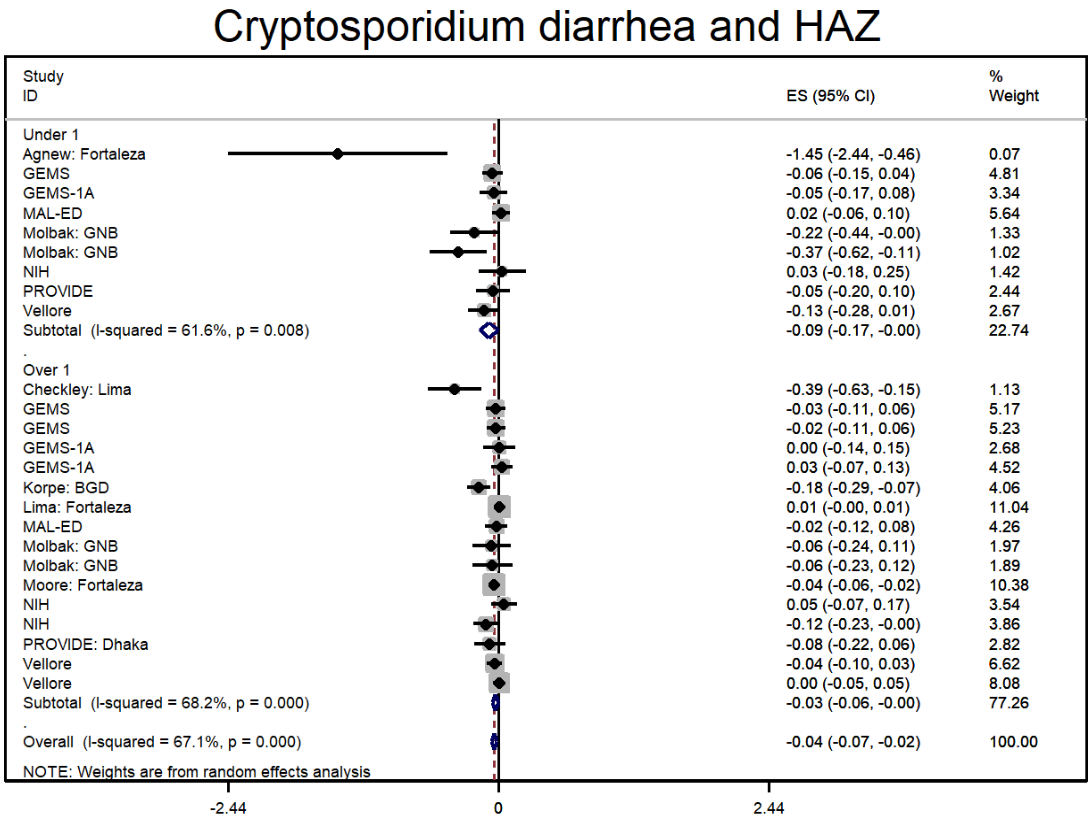

B)

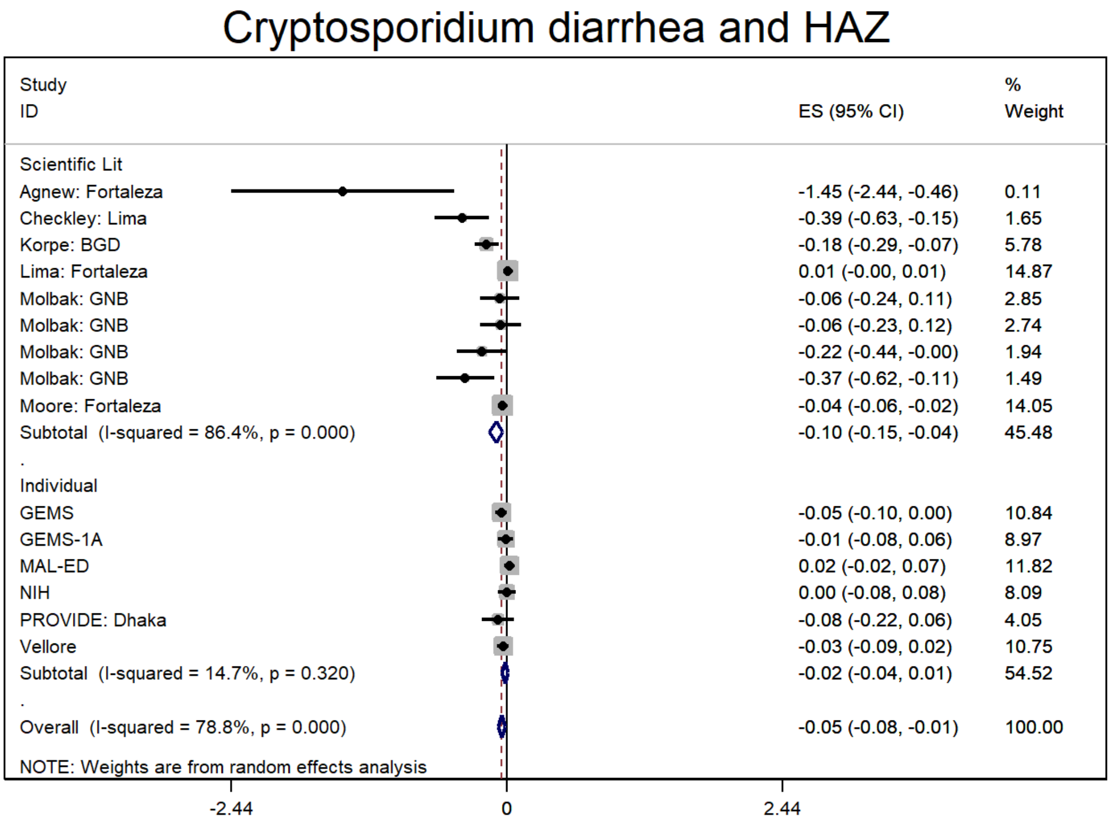
Cryptosporidium diarrhea and HAZ
Study
ID
ES (95% CI)
%
Weight
Scientific Lit
Agnew: Fortaleza -1.45 (-2.44, -0.46) 0.11
Checkley: Lima -0.39 (-0.63, -0.15) 1.65
Korpe: BGD -0.18 (-0.29, -0.07) 5.78
Lima: Fortaleza 0.01 (-0.00, 0.01) 14.87
Molbak: GNB -0.06 (-0.24, 0.11) 2.85
Molbak: GNB -0.06 (-0.23, 0.12) 2.74
Molbak: GNB -0.22 (-0.44, -0.00) 1.94
Molbak: GNB -0.37 (-0.62, -0.11) 1.49
Moore: Fortaleza -0.04 (-0.06, -0.02) 14.05
Subtotal (I-squared = 86.4%, p = 0.000) -0.10 (-0.15, -0.04) 45.48
.
Individual
GEMS -0.05 (-0.10, 0.00) 10.84
GEMS-1A -0.01 (-0.08, 0.06) 8.97
MAL-ED 0.02 (-0.02, 0.07) 11.82
NIH 0.00 (-0.08, 0.08) 8.09
PROVIDE: Dhaka -0.08 (-0.22, 0.06) 4.05
Vellore -0.03 (-0.09, 0.02) 10.75
Subtotal (I-squared = 14.7%, p = 0.320) -0.02 (-0.04, 0.01) 54.52
.
Overall (I-squared = 78.8%, p = 0.000) -0.05 (-0.08, -0.01) 100.00
NOTE: Weights are from random effects analysis
-2.44
0
2.44

C)

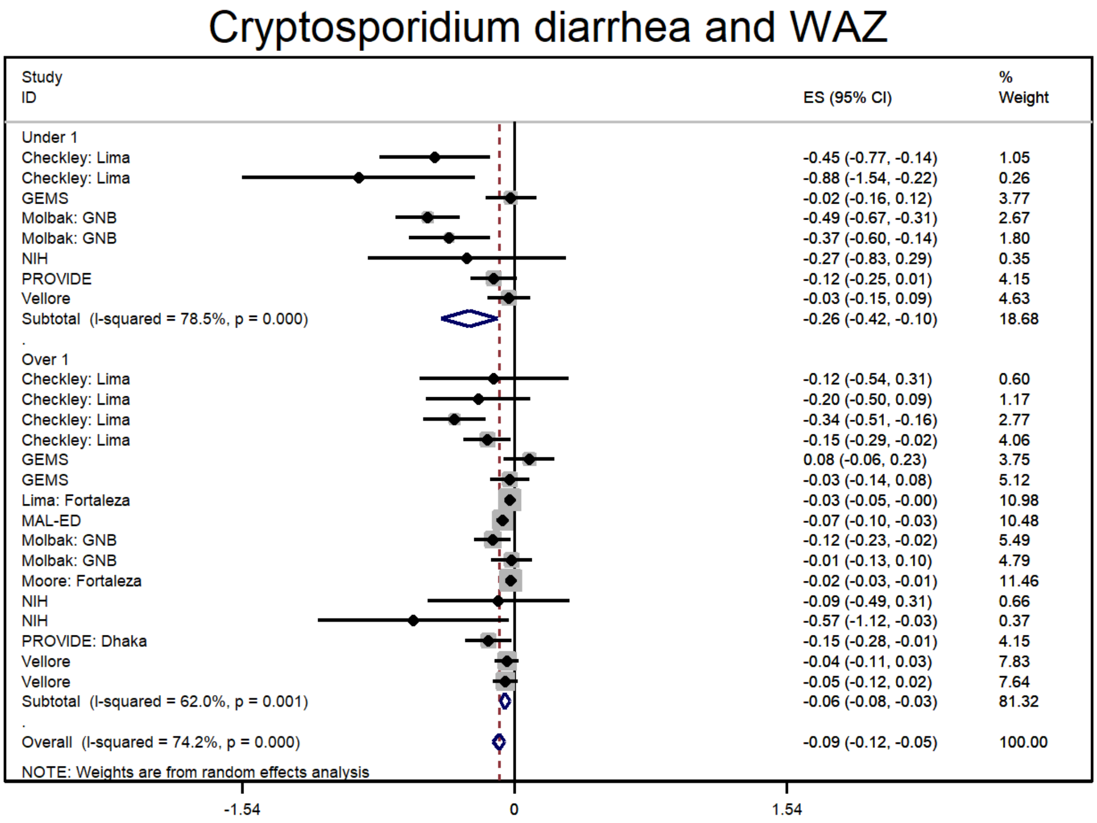
Cryptosporidium diarrhea and WAZ
Study ID
ES (95% CI)
% Weight
Under 1
Checkley: Lima -0.45 (-0.77, -0.14) 1.05
Checkley: Lima -0.88 (-1.54, -0.22) 0.26
GEMS -0.02 (-0.16, 0.12) 3.77
Molbak: GNB -0.49 (-0.67, -0.31) 2.67
Molbak: GNB -0.37 (-0.60, -0.14) 1.80
NIH -0.27 (-0.83, 0.29) 0.35
PROVIDE -0.12 (-0.25, 0.01) 4.15
Vellore -0.03 (-0.15, 0.09) 4.63
Subtotal (I-squared = 78.5%, p = 0.000) -0.26 (-0.42, -0.10) 18.68
Over 1
Checkley: Lima -0.12 (-0.54, 0.31) 0.60
Checkley: Lima -0.20 (-0.50, 0.09) 1.17
Checkley: Lima -0.34 (-0.51, -0.16) 2.77
Checkley: Lima -0.15 (-0.29, -0.02) 4.06
GEMS 0.08 (-0.06, 0.23) 3.75
GEMS -0.03 (-0.14, 0.08) 5.12
Lima: Fortaleza -0.03 (-0.05, -0.00) 10.98
MAL-ED -0.07 (-0.10, -0.03) 10.48
Molbak: GNB -0.12 (-0.23, -0.02) 5.49
Molbak: GNB -0.01 (-0.13, 0.10) 4.79
Moore: Fortaleza -0.02 (-0.03, -0.01) 11.46
NIH -0.09 (-0.49, 0.31) 0.66
NIH -0.57 (-1.12, -0.03) 0.37
PROVIDE: Dhaka -0.15 (-0.28, -0.01) 4.15
Vellore -0.04 (-0.11, 0.03) 7.83
Vellore -0.05 (-0.12, 0.02) 7.64
Subtotal (I-squared = 62.0%, p = 0.001) -0.06 (-0.08, -0.03) 81.32
Overall (I-squared = 74.2%, p = 0.000) -0.09 (-0.12, -0.05) 100.00
NOTE: Weights are from random effects analysis
-1.54
0
1.54

D)

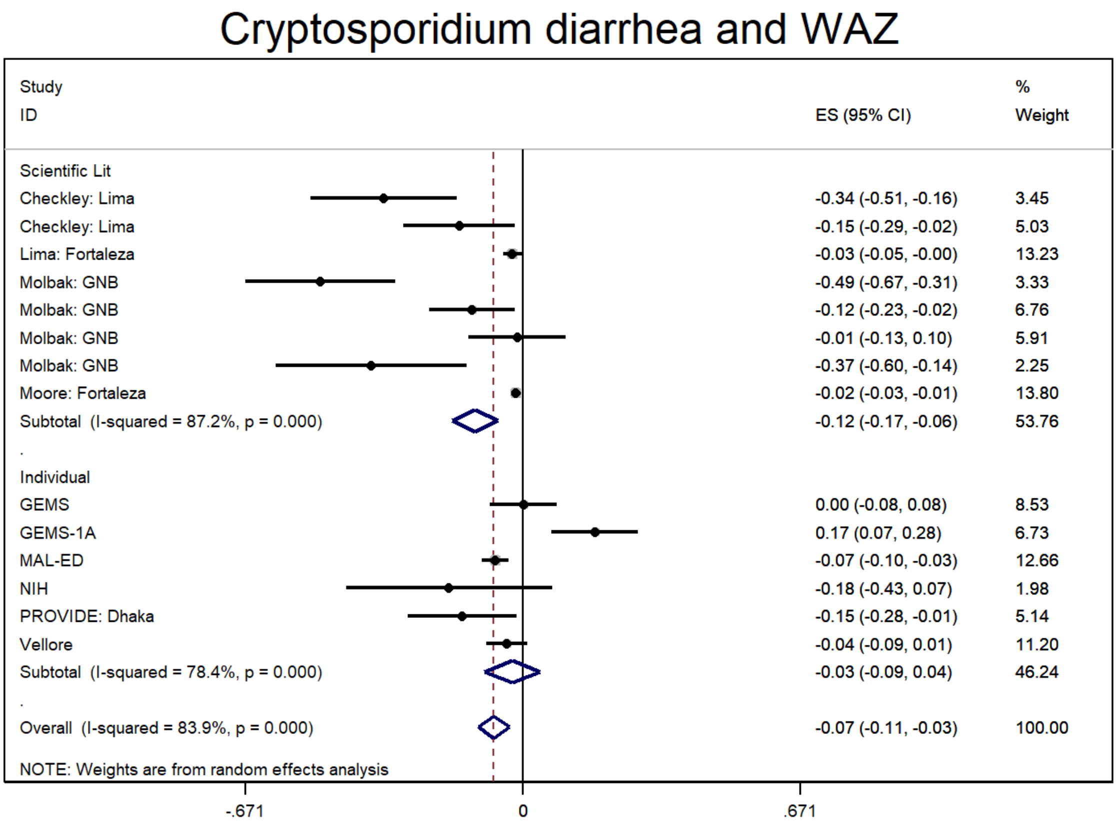

Undernutrition is a risk factor for diarrhoea, measles, and lower respiratory infections (LRI) in GBD 2016, based on the statistically significant relative risks from a systematic review.24,25 Each of these outcomes has a relative risk given undernutrition status (stunted, underweight, wasted) and the relative risks are adjusted for covariance between undernutrition indicators.24 We performed a log-linear interpolation of the reported relative risks at each level of undernutrition reported in the original study (severe, moderate, and mild, stunting, wasting, and underweight) for each outcome. This created a continuous relative risk curve for the increased risk of diarrhoea, LRIs, and measles for each undernutrition indicator.26 Undernutrition was then attributed to a fraction of the DALYs from each of the outcomes based on a counter-factual proportion called the population attributable fraction (PAF).

To determine the number of undernutrition-associated DALYs attributable to Cryptosporidium, we calculated the change, at the population level, in mean HAZ, WAZ, and WHZ due to the pathogen for each age group, year, sex, and geography from GBD. This PAF is defined as:

$$PAF = 1 - \frac{1}{Diarrhea\ Episodes\ *\ Proportion_{Crypto} * \left(\frac{\Delta Zscore}{Crypto\ diarrhea\ episode} \right) * RR}$$

Where the Diarrhoea Episodes is the modelled number of diarrhoea episodes (GBD 2016),27 Proportion Crypto is the frequency of detection of Cryptosporidium in diarrhoea cases (GBD 2016)28, $\Delta Zscore$ is the change in z-score per Cryptosporidium diarrhoea episode from the meta-analysis, and RR is the relative risk of a given outcome (e.g. measles) per z-score change in malnutrition category (i.e. per HAZ, WHZ, or WAZ unit change).24 We did not calculate an undernutrition PAF for children under one month old as neonatal weight is predominantly related to birthweight.

To get a final PAF for undernutrition due to Cryptosporidium diarrhoea, we accounted for covariance in WHZ, WAZ, and HAZ. We used the same approach as the risk factor analysis for undernutrition in GBD 2016 which is defined by:25

$$PAF_{Undernutrition} = 1 - [(1 - PAF_{WAZ}) * (1 - PAF_{HAZ}) * (1 - PAF_{WHZ})]$$

The last step was to multiply our PAF by the LRI, measles, and diarrhoea DALYs estimates to determine the total number of DALYs from those outcomes attributable to Cryptosporidium diarrhoea.

Cryptosporidium affects weight gain. Protein-energy malnutrition (PEM) is a burden of disease that is due to low weight. To estimate the amount of PEM that was due to Cryptosporidium, we estimated the shift in the weight-for-age and weight-for-height distribution due to Cryptosporidium diarrhoea. This was done by evaluating the percent difference in the observed WAZ and WHZ distribution. The shift in the mean WAZ and WHZ at the population level was represented by:

$$Shift = Diarrhea\ Episodes * Proportion_{Crypto} * \left(\frac{\Delta Zscore}{Crypto\ diarrhea\ episode} \right)$$

And the counterfactual prevalence of wasting and underweight was:

$$Prevalence(Counterfactual) = Prevalence(GBD\ 2016\ Estimate) + Shift$$

The prevalence of underweight and wasting was converted to a z-score, and we estimated the percent change in the cumulative density from a normal distribution compared to the observed prevalence.

$$PAF = \frac{Prevalence(GBD\ 2016\ Estimate) - Prevalence\ (Counterfactual)}{Prevalence\ (GBD\ 2016\ Estimate)}$$

PAFs were calculated independently for mild, moderate, and severe undernutrition, age group, geography, sex, and year. Final DALYs due to Cryptosporidium diarrhoea are the PAF multiplied by the number of DALYs due to protein-energy malnutrition.

Supplementary Table 3. Cryptosporidium deaths, incidence, cases, DALYs among children under 5 in 2016 by country

The burden of *Cryptosporidium* by country is shown. The number of deaths, episodes, acute, undernutrition, and total DALYs due to *Cryptosporidium* are accompanied by the incidence per 1,000 child-years and the percent increase in the total number of DALYs after accounting for the long-term outcomes associated with *Cryptosporidium* diarrhoea. Numbers in parentheses are the 95% Uncertainty Intervals

Location	Deaths	Incidence per 1,000	Episodes	Acute DALYs	Undernutrition-associated DALYs	Total DALYs	Percent increase
**Global**	48,301 (24,612 to 81,934)	69.7 (31.1 to 137.9)	44,843,579 (19,989,536 to 88,698,030)	4,223,986 (2,159,974 to 7,163,041)	7,851,321.0 (5,420,601.5 to 11,052,124.5)	12,868,494 (10,148,099 to 16,010,060)	153% (103 to 232%)
**High-income**	3 (1 to 7)	3.1 (0.3 to 10.6)	180,387 (19,662 to 613,939)	571 (98 to 1,550)	2,475.8 (1,547.1 to 3,794.0)	3,776 (2,550 to 5,808)	205% (75 to 422%)
**High-income North America**	0 (0 to 0)	0.2 (0.1 to 0.3)	4,554 (2,706 to 6,555)	31 (25 to 37)	288.1 (194.0 to 409.9)	319 (226 to 442)	910% (614 to 1,353%)
**Canada**	0 (0 to 0)	0.1 (0.1 to 0.2)	278 (162 to 411)	1 (1 to 2)	10.3 (6.4 to 15.4)	12 (8 to 17)	814% (471 to 1,297%)
**Greenland**	0 (0 to 0)	0.2 (0.1 to 0.3)	1 (0 to 1)	0 (0 to 0)	0.1 (0.1 to 0.1)	0 (0 to 0)	1,562% (661 to 2,970%)
**United States**	0 (0 to 0)	0.2 (0.1 to 0.3)	4,276 (2,544 to 6,145)	30 (26 to 35)	277.7 (186.6 to 396.8)	308 (217 to 428)	932% (615 to 1,368%)
**Australasia**	0 (0 to 0)	0.2 (0.0 to 1.0)	272 (76 to 1,769)	6 (1 to 27)	15.8 (10.2 to 23.7)	27 (16 to 51)	180% (45 to 565%)
**Australia**	0 (0 to 0)	0.1 (0.0 to 0.9)	198 (54 to 1,303)	9 (2 to 30)	10.6 (6.6 to 16.2)	19 (11 to 43)	207% (33 to 617%)
**New Zealand**	0 (0 to 0)	0.3 (0.1 to 1.6)	75 (21 to 475)	2 (1 to 8)	5.2 (3.2 to 8.0)	7 (5 to 13)	376% (58 to 995%)
**High-income Asia Pacific**	0 (0 to 0)	0.1 (0.1 to 0.2)	805 (439 to 1,359)	6 (4 to 27)	63.3 (37.8 to 96.5)	71 (46 to 105)	863% (398 to 1,596%)
**Brunei**	0 (0 to 0)	0.1 (0.0 to 0.6)	4 (2 to 21)	0 (0 to 0)	0.8 (0.3 to 1.6)	1 (0 to 2)	834% (151 to 2,477%)
**Japan**	0 (0 to 0)	0.1 (0.1 to 0.2)	583 (328 to 850)	5 (3 to 10)	56.0 (34.0 to 85.5)	61 (40 to 92)	1,142% (450 to 2,178%)
**Singapore**	0 (0 to 0)	0.3 (0.1 to 2.2)	62 (10 to 387)	1 (0 to 3)	3.0 (1.7 to 4.9)	4 (2 to 7)	428% (76 to 1,184%)
**South Korea**	0 (0 to 0)	0.1 (0.0 to 0.1)	156 (90 to 237)	1 (1 to 1)	3.5 (1.8 to 6.0)	4 (3 to 7)	376% (180 to 678%)
**Western Europe**	2 (0 to 6)	7.8 (0.6 to 27.6)	174,157 (12,306 to 613,882)	514 (51 to 1,469)	1,193.9 (627.8 to 2,049.6)	2,429 (1,510 to 4,224)	102% (34 to 237%)
**Andorra**	0 (0 to 0)	15.8 (0.3 to 68.1)	47 (1 to 204)	0 (0 to 2)	0.3 (0.1 to 0.6)	1 (0 to 2)	122% (12 to 467%)
**Austria**	0 (0 to 0)	39.5 (4.4 to 109.8)	15,572 (1,718 to 43,317)	73 (19 to 252)	52.5 (28.1 to 92.4)	125 (61 to 311)	109% (19 to 293%)
**Belgium**	0 (0 to 1)	19.6 (0.3 to 83.1)	12,176 (210 to 51,619)	126 (18 to 466)	70.8 (26.6 to 142.3)	196 (64 to 574)	115% (11 to 447%)
**Cyprus**	0 (0 to 0)	20.1 (0.3 to 85.9)	1,038 (18 to 4,440)	15 (2 to 68)	6.6 (2.6 to 13.1)	22 (7 to 76)	86% (7 to 324%)

Location	Deaths	Incidence per 1,000	Episodes	Acute DALYs	Undernutrition-associated DALYs	Total DALYs	Percent increase
**Denmark**	0<br>(0 to 0)	18.9<br>(0.3 to 78.9)	5,390<br>(94 to 22,550)	59<br>(10 to 199)	28.6<br>(11.1 to 57.0)	87<br>(30 to 226)	89%<br>(11 to 315%)
**Finland**	0<br>(0 to 0)	25.3<br>(1.3 to 84.2)	7,368<br>(383 to 24,547)	32<br>(7 to 117)	33.3<br>(16.7 to 58.9)	65<br>(31 to 153)	172%<br>(23 to 582%)
**France**	0<br>(0 to 0)	0.3<br>(0.2 to 0.5)	1,324<br>(713 to 2,084)	5<br>(4 to 7)	102.9<br>(59.4 to 169.5)	108<br>(64 to 174)	2,028%<br>(1,059 to 3,699%)
**Germany**	1<br>(0 to 2)	17.0<br>(0.4 to 67.7)	59,027<br>(1,227 to 235,205)	444<br>(81 to 1,707)	339.5<br>(168.4 to 638.8)	784<br>(337 to 2,081)	139%<br>(17 to 454%)
**Greece**	0<br>(0 to 0)	18.0<br>(0.7 to 58.7)	8,557<br>(353 to 27,932)	44<br>(11 to 137)	45.4<br>(23.1 to 79.2)	90<br>(44 to 178)	167%<br>(29 to 475%)
**Iceland**	0<br>(0 to 0)	50.0<br>(14.1 to 122.5)	1,069<br>(302 to 2,620)	3<br>(2 to 6)	2.9<br>(1.6 to 4.9)	6<br>(4 to 9)	95%<br>(41 to 199%)
**Ireland**	0<br>(0 to 0)	3.0<br>(0.2 to 19.3)	1,129<br>(82 to 7,232)	9<br>(2 to 27)	17.1<br>(7.8 to 32.2)	26<br>(12 to 49)	311%<br>(49 to 948%)
**Israel**	0<br>(0 to 0)	0.2<br>(0.1 to 0.4)	199<br>(105 to 316)	1<br>(1 to 3)	18.6<br>(11.0 to 29.6)	20<br>(12 to 31)	1,482%<br>(546 to 2,911%)
**Italy**	0<br>(0 to 0)	0.2<br>(0.1 to 0.5)	656<br>(323 to 1,422)	4<br>(2 to 8)	49.8<br>(28.3 to 80.1)	53<br>(31 to 85)	1,609%<br>(542 to 3,568%)
**Luxembourg**	0<br>(0 to 0)	18.2<br>(0.3 to 74.8)	529<br>(9 to 2,179)	6<br>(1 to 25)	2.5<br>(0.9 to 5.0)	9<br>(3 to 28)	79%<br>(8 to 268%)
**Malta**	0<br>(0 to 0)	6.7<br>(0.1 to 29.1)	142<br>(2 to 619)	2<br>(0 to 6)	0.8<br>(0.3 to 1.5)	2<br>(1 to 6)	88%<br>(10 to 316%)
**Netherlands**	0<br>(0 to 1)	34.6<br>(0.6 to 127.2)	30,102<br>(481 to 110,708)	215<br>(33 to 788)	123.6<br>(54.7 to 234.3)	339<br>(127 to 932)	111%<br>(12 to 417%)
**Norway**	0<br>(0 to 0)	15.1<br>(0.3 to 63.6)	4,286<br>(75 to 18,003)	31<br>(6 to 109)	18.0<br>(6.6 to 36.0)	49<br>(17 to 127)	100%<br>(12 to 331%)
**Portugal**	0<br>(0 to 0)	0.2<br>(0.1 to 0.4)	112<br>(60 to 170)	0<br>(0 to 1)	6.8<br>(3.6 to 11.5)	7<br>(4 to 12)	1,643%<br>(841 to 2,880%)
**Spain**	0<br>(0 to 0)	0.3<br>(0.1 to 0.4)	636<br>(337 to 1,000)	2<br>(1 to 2)	31.3<br>(18.3 to 49.9)	33<br>(20 to 52)	1,757%<br>(933 to 3,003%)
**Sweden**	0<br>(0 to 0)	15.3<br>(0.5 to 55.3)	8,701<br>(264 to 31,429)	49<br>(11 to 152)	47.3<br>(25.0 to 82.2)	97<br>(46 to 204)	149%<br>(27 to 432%)
**Switzerland**	0<br>(0 to 0)	5.2<br>(0.3 to 27.8)	2,157<br>(137 to 11,525)	21<br>(6 to 60)	30.7<br>(15.5 to 55.0)	52<br>(27 to 99)	213%<br>(38 to 608%)
**England**	0<br>(0 to 0)	0.3<br>(0.2 to 0.5)	1,097<br>(596 to 1,686)	3<br>(3 to 4)	101.9<br>(60.0 to 153.3)	105<br>(63 to 156)	3,043%<br>(1,663 to 4,886%)
**Northern Ireland**	0<br>(0 to 0)	0.4<br>(0.2 to 1.0)	48<br>(24 to 117)	0<br>(0 to 1)	5.8<br>(2.9 to 10.3)	6<br>(3 to 11)	1,849%<br>(512 to 4,617%)
**Scotland**	0<br>(0 to 0)	0.4<br>(0.2 to 0.6)	99<br>(52 to 155)	0<br>(0 to 0)	5.7<br>(3.0 to 10.0)	6<br>(3 to 10)	1,653%<br>(796 to 2,999%)
**Wales**	0<br>(0 to 1)	85.0<br>(7.7 to 236.0)	13,676<br>(1,233 to 37,954)	88<br>(30 to 240)	51.1<br>(22.8 to 91.4)	139<br>(69 to 293)	78%<br>(15 to 215%)
**Southern Latin America**	0<br>(0 to 0)	0.6<br>(0.3 to 0.9)	2,966<br>(1,620 to 4,477)	14<br>(10 to 32)	914.7<br>(588.1 to 1,376.7)	930<br>(602 to 1,393)	5,891%<br>(3,524 to 9,808%)
**Argentina**	0<br>(0 to 0)	0.7<br>(0.4 to 1.0)	2,380<br>(1,302 to 3,604)	13<br>(9 to 18)	820.8<br>(522.2 to 1,256.3)	834<br>(534 to 1,271)	6,522%<br>(3,592 to 10,919%)

Location	Deaths	Incidence per 1,000	Episodes	Acute DALYs	Undernutrition-associated DALYs	Total DALYs	Percent increase
**Chile**	0<br>(0 to 0)	0.4<br>(0.2 to 0.7)	531<br>(293 to 800)	2<br>(1 to 2)	74.2<br>(44.7 to 117.9)	76<br>(46 to 120)	4,665%<br>(2,558 to 7,698%)
**Uruguay**	0<br>(0 to 0)	0.2<br>(0.1 to 0.4)	55<br>(30 to 86)	1<br>(0 to 1)	19.6<br>(10.5 to 34.2)	20<br>(11 to 35)	3,166%<br>(1,451 to 6,051%)
**Central Europe, Eastern Europe, and Central Asia**	32<br>(2 to 93)	12.5<br>(1.0 to 40.8)	381,279<br>(29,096 to 1,241,514)	3,457<br>(247 to 9,896)	58,479.0<br>(38,887.1 to 82,597.2)	65,998<br>(45,021 to 91,974)	903%<br>(291 to 1,802%)
**Eastern Europe**	5<br>(0 to 12)	21.0<br>(1.2 to 62.5)	287,895<br>(15,793 to 855,817)	987<br>(115 to 2,372)	9,294.7<br>(6,453.6 to 13,280.9)	11,102<br>(7,735 to 16,313)	590%<br>(195 to 1,295%)
**Belarus**	0<br>(0 to 0)	19.5<br>(1.0 to 65.2)	11,617<br>(576 to 38,933)	51<br>(14 to 142)	175.9<br>(105.8 to 289.1)	227<br>(138 to 369)	496%<br>(106 to 1,431%)
**Estonia**	0<br>(0 to 0)	34.8<br>(1.8 to 106.9)	2,669<br>(140 to 8,208)	11<br>(3 to 35)	62.4<br>(39.3 to 93.7)	73<br>(45 to 112)	893%<br>(176 to 2,449%)
**Latvia**	0<br>(0 to 0)	30.5<br>(1.5 to 98.5)	3,945<br>(198 to 12,730)	16<br>(4 to 43)	79.8<br>(46.8 to 123.6)	96<br>(58 to 147)	714%<br>(159 to 1,922%)
**Lithuania**	0<br>(0 to 0)	34.6<br>(1.8 to 114.6)	6,104<br>(313 to 20,245)	27<br>(7 to 81)	145.0<br>(90.3 to 221.0)	172<br>(108 to 262)	784%<br>(177 to 2,271%)
**Moldova**	0<br>(0 to 0)	19.2<br>(1.0 to 59.1)	4,285<br>(225 to 13,166)	39<br>(13 to 102)	217.8<br>(121.8 to 368.8)	257<br>(155 to 412)	728%<br>(182 to 1,882%)
**Russia**	4<br>(0 to 11)	19.7<br>(1.0 to 59.4)	195,134<br>(10,053 to 587,250)	1,341<br>(360 to 4,151)	6,994.5<br>(4,758.2 to 10,249.1)	8,337<br>(5,650 to 12,150)	746%<br>(173 to 1,967%)
**Ukraine**	1<br>(0 to 2)	24.7<br>(1.2 to 78.7)	64,144<br>(3,222 to 204,800)	320<br>(93 to 873)	1,619.3<br>(929.1 to 2,698.2)	1,940<br>(1,145 to 3,297)	697%<br>(170 to 1,874%)
**Central Europe**	1<br>(0 to 2)	4.2<br>(0.8 to 23.4)	23,994<br>(4,618 to 133,721)	99<br>(14 to 390)	4,448.2<br>(2,860.9 to 6,512.7)	4,619<br>(3,112 to 6,694)	2,797%<br>(1,110 to 5,218%)
**Albania**	0<br>(0 to 0)	3.6<br>(0.6 to 21.1)	622<br>(108 to 3,661)	4<br>(1 to 13)	239.3<br>(117.4 to 423.1)	243<br>(122 to 426)	8,227%<br>(1,462 to 23,398%)
**Bosnia and Herzegovina**	0<br>(0 to 0)	3.0<br>(0.5 to 17.8)	526<br>(94 to 3,070)	6<br>(2 to 15)	50.3<br>(30.3 to 78.4)	56<br>(34 to 87)	1,282%<br>(291 to 3,330%)
**Bulgaria**	0<br>(0 to 0)	3.7<br>(0.7 to 21.0)	1,248<br>(230 to 7,132)	14<br>(4 to 50)	342.6<br>(176.8 to 606.7)	357<br>(189 to 627)	3,801%<br>(567 to 11,659%)
**Croatia**	0<br>(0 to 0)	4.4<br>(0.9 to 23.6)	879<br>(179 to 4,701)	4<br>(1 to 10)	68.6<br>(42.2 to 106.7)	72<br>(45 to 110)	2,508%<br>(615 to 6,629%)
**Czech Republic**	0<br>(0 to 0)	4.0<br>(0.8 to 23.1)	2,285<br>(449 to 13,072)	11<br>(4 to 27)	157.9<br>(99.1 to 250.4)	168<br>(107 to 266)	1,987%<br>(534 to 4,888%)
**Hungary**	0<br>(0 to 0)	3.5<br>(0.7 to 20.3)	1,795<br>(336 to 10,345)	10<br>(3 to 27)	166.2<br>(94.3 to 269.4)	176<br>(106 to 280)	2,169%<br>(521 to 5,477%)
**Macedonia**	0<br>(0 to 0)	3.3<br>(0.6 to 18.4)	374<br>(71 to 2,093)	5<br>(1 to 18)	70.8<br>(41.0 to 118.1)	76<br>(45 to 129)	2,062%<br>(340 to 5,801%)
**Montenegro**	0<br>(0 to 0)	2.2<br>(0.4 to 12.4)	81<br>(15 to 458)	1<br>(0 to 2)	8.2<br>(4.7 to 13.1)	9<br>(5 to 14)	2,066%<br>(413 to 5,798%)
**Poland**	0<br>(0 to 0)	4.4<br>(0.8 to 25.5)	8,686<br>(1,617 to 50,710)	32<br>(11 to 81)	760.6<br>(415.9 to 1,237.2)	793<br>(453 to 1,266)	3,024%<br>(822 to 7,521%)
**Romania**	0<br>(0 to 1)	5.1<br>(1.0 to 30.8)	4,026<br>(773 to 24,562)	63<br>(15 to 229)	2,136.4<br>(1,285.6 to 3,343.9)	2,199<br>(1,352 to 3,392)	5,527%<br>(804 to 16,218%)

Location	Deaths	Incidence per 1,000	Episodes	Acute DALYs	Undernutrition-associated DALYs	Total DALYs	Percent increase
**Serbia**	0<br>(0 to 0)	3.9<br>(0.8 to 20.4)	1,598<br>(322 to 8,358)	11<br>(4 to 32)	209.0<br>(133.3 to 303.0)	220<br>(142 to 318)	2,549%<br>(628 to 6,235%)
**Slovakia**	0<br>(0 to 0)	4.6<br>(0.8 to 26.9)	1,363<br>(252 to 7,997)	8<br>(3 to 24)	201.8<br>(111.7 to 313.6)	210<br>(121 to 323)	3,290%<br>(791 to 8,677%)
**Slovenia**	0<br>(0 to 0)	4.8<br>(0.9 to 27.6)	510<br>(95 to 2,934)	2<br>(1 to 4)	36.5<br>(20.2 to 59.1)	38<br>(21 to 61)	2,739%<br>(701 to 6,912%)
**Central Asia**	26<br>(1 to 79)	6.3<br>(0.5 to 27.6)	68,917<br>(5,206 to 304,412)	2,371<br>(91 to 7,278)	44,736.2<br>(28,312.0 to 65,267.0)	50,277<br>(32,921 to 72,541)	982%<br>(252 to 2,434%)
**Armenia**	0<br>(0 to 0)	8.2<br>(0.6 to 36.5)	1,852<br>(141 to 8,219)	29<br>(7 to 102)	394.8<br>(220.8 to 638.4)	424<br>(250 to 670)	2,114%<br>(351 to 5,705%)
**Azerbaijan**	3<br>(0 to 9)	10.6<br>(0.8 to 46.3)	9,851<br>(726 to 42,895)	598<br>(119 to 2,297)	6,944.7<br>(3,354.4 to 12,201.6)	7,544<br>(3,842 to 13,079)	2,063%<br>(274 to 6,608%)
**Georgia**	0<br>(0 to 0)	8.0<br>(0.6 to 37.1)	2,234<br>(170 to 10,370)	34<br>(9 to 115)	195.7<br>(110.6 to 322.0)	230<br>(132 to 382)	837%<br>(163 to 2,297%)
**Kazakhstan**	1<br>(0 to 3)	5.6<br>(0.4 to 25.3)	10,843<br>(835 to 49,492)	195<br>(49 to 662)	1,375.8<br>(771.3 to 2,258.7)	1,571<br>(900 to 2,528)	1,079%<br>(200 to 3,150%)
**Kyrgyzstan**	3<br>(0 to 9)	6.3<br>(0.5 to 27.2)	5,110<br>(395 to 22,060)	541<br>(103 to 1,943)	2,913.0<br>(1,846.5 to 4,452.4)	3,454<br>(2,169 to 5,578)	964%<br>(132 to 2,922%)
**Mongolia**	0<br>(0 to 0)	7.2<br>(0.6 to 34.4)	2,688<br>(217 to 12,789)	14<br>(4 to 43)	1,011.9<br>(573.8 to 1,683.7)	1,026<br>(592 to 1,690)	10,403%<br>(2,035 to 28,958%)
**Tajikistan**	16<br>(0 to 52)	12.1<br>(0.9 to 52.8)	13,310<br>(971 to 57,932)	3,443<br>(579 to 12,839)	16,159.4<br>(8,341.3 to 27,654.2)	19,603<br>(10,240 to 33,051)	915%<br>(112 to 3,057%)
**Turkmenistan**	2<br>(0 to 5)	12.1<br>(0.9 to 53.6)	6,493<br>(482 to 28,730)	298<br>(64 to 1,090)	5,021.0<br>(2,683.9 to 8,650.4)	5,319<br>(2,882 to 9,092)	2,671%<br>(433 to 8,095%)
**Uzbekistan**	2<br>(0 to 6)	3.4<br>(0.3 to 15.7)	16,472<br>(1,207 to 75,363)	386<br>(97 to 1,257)	10,720.0<br>(4,757.7 to 20,359.8)	11,106<br>(5,145 to 21,096)	4,234%<br>(643 to 12,017%)
**Latin America and Caribbean**	278<br>(101 to 547)	76.0<br>(32.4 to 150.0)	3,592,415<br>(1,534,545 to 7,091,909)	30,316<br>(13,003 to 56,532)	192,416.7<br>(141,033.7 to 257,872.2)	231,571<br>(175,793 to 302,160)	506%<br>(291 to 788%)
**Central Latin America**	11<br>(3 to 32)	2.3<br>(0.8 to 6.1)	52,473<br>(17,702 to 136,283)	1,064<br>(293 to 2,969)	23,969.3<br>(16,118.6 to 33,131.1)	25,606<br>(17,623 to 35,172)	1,636%<br>(597 to 3,376%)
**Colombia**	0<br>(0 to 2)	1.2<br>(0.5 to 5.2)	4,006<br>(1,685 to 16,688)	54<br>(21 to 139)	2,037.1<br>(1,006.2 to 3,606.0)	2,091<br>(1,056 to 3,663)	4,728%<br>(1,173 to 11,997%)
**Costa Rica**	0<br>(0 to 0)	1.4<br>(0.6 to 6.1)	470<br>(196 to 1,967)	4<br>(1 to 10)	111.2<br>(50.3 to 208.7)	115<br>(53 to 214)	3,867%<br>(912 to 10,526%)
**El Salvador**	0<br>(0 to 1)	1.9<br>(0.8 to 8.5)	879<br>(371 to 4,006)	20<br>(6 to 61)	539.4<br>(256.7 to 1,043.3)	559<br>(275 to 1,056)	3,939%<br>(708 to 11,745%)
**Guatemala**	1<br>(0 to 3)	1.6<br>(0.9 to 2.2)	3,075<br>(1,810 to 4,198)	80<br>(43 to 162)	11,264.1<br>(7,401.5 to 15,983.6)	11,344<br>(7,471 to 16,063)	15,855%<br>(6,272 to 28,108%)
**Honduras**	8<br>(1 to 20)	33.0<br>(2.3 to 98.0)	30,942<br>(2,169 to 91,869)	1,256<br>(400 to 3,335)	2,787.2<br>(1,674.4 to 4,356.0)	4,044<br>(2,505 to 6,380)	298%<br>(72 to 783%)
**Mexico**	1<br>(1 to 8)	0.6<br>(0.3 to 0.9)	6,533<br>(3,399 to 10,240)	164<br>(73 to 364)	5,012.2<br>(3,327.0 to 7,435.7)	5,176<br>(3,471 to 7,597)	3,650%<br>(1,258 to 7,711%)

Location	Deaths	Incidence per 1,000	Episodes	Acute DALYs	Undernutrition-associated DALYs	Total DALYs	Percent increase
**Nicaragua**	0<br>(0 to 0)	1.0<br>(0.6 to 1.5)	630<br>(357 to 912)	8<br>(6 to 11)	656.7<br>(396.7 to 1,007.8)	664<br>(404 to 1,016)	8,798%<br>(4,742 to 14,631%)
**Panama**	0<br>(0 to 1)	1.7<br>(0.7 to 7.0)	629<br>(272 to 2,537)	19<br>(7 to 51)	682.2<br>(331.7 to 1,229.5)	701<br>(351 to 1,245)	4,799%<br>(1,041 to 12,225%)
**Venezuela**	0<br>(0 to 0)	1.6<br>(0.9 to 2.3)	4,447<br>(2,450 to 6,542)	32<br>(25 to 40)	879.3<br>(534.7 to 1,312.8)	911<br>(566 to 1,347)	2,818%<br>(1,626 to 4,574%)
**Andean Latin America**	11<br>(0 to 31)	11.6<br>(0.9 to 46.9)	76,123<br>(5,975 to 307,379)	1,058<br>(56 to 3,042)	22,237.9<br>(15,571.5 to 31,453.4)	24,589<br>(17,470 to 34,027)	1,142%<br>(336 to 2,776%)
**Bolivia**	4<br>(0 to 14)	10.4<br>(1.0 to 53.1)	13,836<br>(1,331 to 70,926)	930<br>(149 to 3,708)	10,250.1<br>(6,277.3 to 15,984.4)	11,181<br>(6,941 to 17,245)	2,251%<br>(270 to 7,677%)
**Ecuador**	2<br>(0 to 6)	8.8<br>(0.9 to 44.6)	14,860<br>(1,511 to 75,326)	367<br>(85 to 1,198)	4,248.7<br>(2,731.3 to 6,262.3)	4,616<br>(3,025 to 6,844)	1,848%<br>(300 to 5,135%)
**Peru**	5<br>(0 to 14)	13.5<br>(0.8 to 49.9)	47,553<br>(2,952 to 176,392)	1,051<br>(218 to 3,469)	7,739.1<br>(4,723.4 to 11,997.3)	8,791<br>(5,583 to 13,503)	1,245%<br>(177 to 3,530%)
**Caribbean**	101<br>(11 to 244)	36.6<br>(1.9 to 107.5)	151,161<br>(7,802 to 443,712)	9,006<br>(1,043 to 21,559)	40,278.1<br>(21,890.4 to 69,604.8)	54,830<br>(32,303 to 89,568)	302%<br>(104 to 783%)
**Antigua and Barbuda**	0<br>(0 to 0)	38.8<br>(1.9 to 122.0)	179<br>(9 to 564)	2<br>(1 to 5)	7.6<br>(4.1 to 12.7)	10<br>(6 to 16)	450%<br>(117 to 1,194%)
**The Bahamas**	0<br>(0 to 0)	32.5<br>(1.6 to 102.6)	1,037<br>(51 to 3,275)	10<br>(4 to 26)	42.4<br>(18.1 to 93.2)	53<br>(26 to 104)	545%<br>(116 to 1,552%)
**Barbados**	0<br>(0 to 0)	30.9<br>(1.5 to 100.1)	484<br>(24 to 1,571)	4<br>(1 to 12)	17.9<br>(8.2 to 36.0)	22<br>(11 to 41)	570%<br>(116 to 1,565%)
**Belize**	0<br>(0 to 0)	36.0<br>(1.9 to 106.6)	1,486<br>(78 to 4,403)	27<br>(9 to 71)	89.0<br>(35.6 to 187.1)	116<br>(56 to 229)	428%<br>(97 to 1,197%)
**Bermuda**	0<br>(0 to 0)	32.7<br>(1.5 to 109.1)	145<br>(7 to 483)	1<br>(0 to 2)	1.8<br>(1.0 to 2.9)	2<br>(1 to 4)	426%<br>(78 to 1,245%)
**Cuba**	0<br>(0 to 1)	19.5<br>(0.9 to 63.5)	12,000<br>(583 to 39,163)	95<br>(31 to 242)	200.9<br>(124.5 to 300.2)	296<br>(192 to 472)	276%<br>(72 to 672%)
**Dominica**	0<br>(0 to 0)	31.9<br>(1.6 to 101.0)	172<br>(9 to 546)	2<br>(1 to 6)	8.1<br>(4.3 to 14.7)	10<br>(6 to 17)	435%<br>(112 to 1,100%)
**Dominican Republic**	8<br>(1 to 20)	37.6<br>(1.9 to 116.0)	38,275<br>(1,916 to 118,039)	1,201<br>(384 to 3,044)	3,636.0<br>(2,041.6 to 6,157.1)	4,838<br>(2,830 to 7,869)	396%<br>(104 to 1,077%)
**Grenada**	0<br>(0 to 0)	29.9<br>(1.4 to 97.8)	357<br>(17 to 1,167)	3<br>(1 to 8)	16.8<br>(8.3 to 31.4)	20<br>(11 to 37)	744%<br>(154 to 2,004%)
**Guyana**	1<br>(0 to 2)	26.3<br>(1.3 to 76.6)	1,875<br>(96 to 5,467)	93<br>(34 to 218)	186.0<br>(108.4 to 293.6)	280<br>(173 to 426)	249%<br>(72 to 588%)
**Haiti**	90<br>(10 to 217)	52.4<br>(2.8 to 149.9)	79,177<br>(4,171 to 226,598)	12,792<br>(4,095 to 32,882)	35,333.8<br>(18,577.9 to 63,315.3)	48,138<br>(26,525 to 81,804)	362%<br>(94 to 965%)
**Jamaica**	1<br>(0 to 2)	21.6<br>(1.1 to 63.8)	5,789<br>(284 to 17,076)	136<br>(44 to 364)	333.3<br>(156.8 to 630.5)	469<br>(248 to 849)	325%<br>(72 to 861%)
**Puerto Rico**	0<br>(0 to 0)	24.5<br>(1.2 to 79.9)	5,898<br>(292 to 19,216)	42<br>(15 to 97)	87.8<br>(54.3 to 138.1)	130<br>(82 to 212)	265%<br>(81 to 596%)

Location	Deaths	Incidence per 1,000	Episodes	Acute DALYs	Undernutrition-associated DALYs	Total DALYs	Percent increase
**Saint Lucia**	0 (0 to 0)	46.1 (2.3 to 142.5)	462 (23 to 1,426)	4 (2 to 12)	17.3 (8.6 to 33.3)	22 (12 to 39)	529% (128 to 1,381%)
**Saint Vincent and the Grenadines**	0 (0 to 0)	28.4 (1.4 to 87.2)	270 (13 to 831)	5 (2 to 14)	15.3 (7.8 to 27.3)	21 (12 to 35)	376% (93 to 998%)
**Suriname**	1 (0 to 2)	32.0 (1.6 to 95.4)	1,686 (84 to 5,035)	92 (27 to 241)	230.5 (111.8 to 418.0)	323 (178 to 552)	341% (77 to 906%)
**Trinidad and Tobago**	0 (0 to 0)	17.9 (0.9 to 54.8)	1,591 (77 to 4,865)	26 (8 to 74)	49.6 (18.9 to 111.1)	75 (35 to 149)	259% (51 to 781%)
**Virgin Islands, U.S.**	0 (0 to 0)	31.5 (1.5 to 105.4)	172 (8 to 575)	1 (0 to 2)	3.8 (2.2 to 6.2)	5 (3 to 7)	553% (141 to 1,313%)
**Tropical Latin America**	155 (81 to 260)	231.6 (103.5 to 429.1)	3,301,955 (1,475,682 to 6,117,901)	19,188 (10,637 to 31,428)	105,931.3 (79,146.0 to 140,758.3)	126,547 (98,929 to 161,784)	517% (327 to 797%)
**Brazil**	147 (78 to 249)	223.6 (99.6 to 415.8)	3,118,354 (1,388,699 to 5,799,030)	19,536 (13,725 to 27,994)	100,095.6 (74,628.8 to 133,872.4)	119,651 (93,166 to 153,293)	529% (319 to 812%)
**Paraguay**	8 (3 to 14)	586.6 (271.0 to 1,053.2)	183,471 (84,760 to 329,441)	1,058 (682 to 1,606)	5,835.7 (3,813.4 to 8,638.0)	6,895 (4,832 to 9,772)	576% (307 to 1,012%)
**Southeast Asia, East Asia, and Oceania**	366 (109 to 864)	12.6 (4.3 to 34.4)	1,585,748 (539,267 to 4,345,851)	34,326 (10,842 to 79,016)	183,582.6 (123,678.6 to 265,109.6)	236,225 (168,189 to 325,059)	368% (159 to 666%)
**East Asia**	185 (93 to 334)	17.5 (7.1 to 38.5)	1,159,312 (470,902 to 2,544,555)	18,044 (9,085 to 32,364)	32,581.4 (21,621.4 to 47,568.7)	52,494 (39,330 to 70,365)	162% (95 to 289%)
**China**	124 (63 to 207)	14.3 (5.8 to 31.7)	882,226 (359,759 to 1,958,819)	13,140 (8,533 to 20,087)	17,488.2 (11,670.4 to 25,304.3)	30,642 (22,604 to 41,333)	139% (76 to 237%)
**North Korea**	60 (23 to 131)	71.3 (27.2 to 150.7)	233,990 (89,408 to 494,941)	6,583 (3,200 to 13,016)	14,880.7 (8,010.0 to 25,698.7)	21,471 (13,002 to 34,134)	256% (93 to 572%)
**Taiwan**	1 (0 to 2)	40.3 (13.9 to 95.3)	42,081 (14,508 to 99,532)	169 (94 to 282)	212.5 (133.7 to 326.9)	381 (267 to 545)	137% (62 to 266%)
**Southeast Asia**	164 (9 to 506)	6.1 (0.6 to 28.4)	357,786 (36,967 to 1,669,495)	14,717 (820 to 46,212)	136,958.4 (89,042.7 to 204,856.5)	167,615 (113,162 to 243,030)	517% (150 to 1,300%)
**Cambodia**	0 (0 to 3)	1.1 (0.6 to 3.2)	2,097 (1,097 to 5,852)	57 (17 to 165)	7,653.1 (4,290.2 to 13,012.5)	7,710 (4,331 to 13,090)	18,162% (3,784 to 46,818%)
**Indonesia**	114 (5 to 318)	7.0 (0.6 to 30.8)	168,907 (15,585 to 740,930)	19,355 (4,299 to 73,786)	71,415.0 (49,343.7 to 102,579.8)	90,789 (60,398 to 142,554)	597% (99 to 1,686%)
**Laos**	9 (0 to 43)	8.3 (0.7 to 46.2)	8,933 (718 to 49,877)	2,414 (331 to 10,421)	13,024.5 (4,612.2 to 28,156.6)	15,411 (5,990 to 32,142)	1,141% (112 to 4,829%)
**Malaysia**	1 (0 to 2)	5.1 (0.4 to 34.2)	13,403 (1,019 to 89,080)	150 (30 to 475)	678.1 (340.8 to 1,202.9)	829 (429 to 1,456)	728% (116 to 2,312%)
**Maldives**	0 (0 to 0)	3.5 (0.3 to 22.3)	129 (10 to 823)	1 (0 to 4)	4.7 (2.3 to 8.6)	6 (3 to 10)	553% (96 to 1,739%)

Location	Deaths	Incidence per 1,000	Episodes	Acute DALYs	Undernutrition-associated DALYs	Total DALYs	Percent increase
**Mauritius**	0<br>(0 to 0)	5.1<br>(0.4 to 31.4)	341<br>(27 to 2,080)	10<br>(2 to 32)	29.9<br>(15.7 to 54.3)	40<br>(22 to 72)	491%<br>(74 to 1,450%)
**Myanmar**	11<br>(0 to 57)	4.8<br>(0.4 to 28.0)	29,932<br>(2,423 to 175,011)	2,342<br>(456 to 8,641)	14,722.9<br>(6,246.6 to 30,603.0)	17,067<br>(7,868 to 34,050)	1,140%<br>(137 to 3,770%)
**Philippines**	25<br>(1 to 106)	5.4<br>(0.4 to 32.1)	62,578<br>(5,112 to 371,791)	5,242<br>(998 to 19,582)	22,134.8<br>(12,104.6 to 37,002.9)	27,374<br>(15,226 to 46,928)	743%<br>(93 to 2,425%)
**Sri Lanka**	1<br>(0 to 2)	21.2<br>(0.6 to 74.9)	29,551<br>(814 to 104,322)	253<br>(51 to 930)	435.8<br>(221.7 to 762.1)	686<br>(338 to 1,388)	288%<br>(34 to 917%)
**Seychelles**	0<br>(0 to 0)	5.1<br>(0.4 to 31.9)	43<br>(3 to 268)	0<br>(0 to 1)	4.1<br>(2.1 to 7.3)	4<br>(2 to 8)	1,538%<br>(307 to 4,358%)
**Thailand**	0<br>(0 to 0)	0.7<br>(0.4 to 1.1)	1,814<br>(1,029 to 2,696)	8<br>(6 to 11)	444.2<br>(263.5 to 731.0)	452<br>(272 to 740)	5,657%<br>(3,108 to 9,784%)
**Timor-Leste**	2<br>(0 to 10)	11.3<br>(0.9 to 64.6)	1,854<br>(146 to 10,628)	521<br>(80 to 2,200)	1,554.4<br>(593.1 to 3,153.2)	2,051<br>(820 to 4,212)	602%<br>(47 to 2,297%)
**Vietnam**	1<br>(0 to 5)	5.4<br>(0.4 to 33.7)	38,868<br>(3,145 to 240,876)	338<br>(80 to 1,105)	4,857.0<br>(2,409.8 to 8,755.3)	5,195<br>(2,737 to 9,047)	2,253%<br>(399 to 6,827%)
**Oceania**	17<br>(5 to 38)	47.5<br>(11.6 to 114.2)	65,568<br>(16,037 to 157,718)	1,565<br>(439 to 3,508)	14,042.8<br>(6,994.1 to 26,627.3)	16,116<br>(9,085 to 28,598)	689%<br>(259 to 1,644%)
**American Samoa**	0<br>(0 to 0)	34.2<br>(7.5 to 84.0)	256<br>(56 to 628)	1<br>(1 to 3)	5.5<br>(3.5 to 8.2)	7<br>(5 to 10)	477%<br>(185 to 998%)
**Federated States of Micronesia**	0<br>(0 to 0)	30.7<br>(7.0 to 75.0)	304<br>(69 to 741)	2<br>(1 to 4)	13.7<br>(6.5 to 24.8)	16<br>(9 to 27)	697%<br>(220 to 1,570%)
**Fiji**	1<br>(0 to 1)	37.0<br>(9.0 to 89.6)	2,013<br>(490 to 4,878)	72<br>(33 to 151)	305.0<br>(152.8 to 542.6)	377<br>(213 to 630)	492%<br>(167 to 1,138%)
**Guam**	0<br>(0 to 0)	35.0<br>(7.3 to 87.3)	611<br>(128 to 1,525)	2<br>(1 to 4)	19.7<br>(11.6 to 31.6)	22<br>(13 to 34)	1,067%<br>(404 to 2,170%)
**Kiribati**	0<br>(0 to 1)	48.4<br>(11.8 to 119.0)	658<br>(160 to 1,617)	34<br>(16 to 70)	143.8<br>(66.3 to 263.1)	178<br>(97 to 306)	484%<br>(150 to 1,167%)
**Marshall Islands**	0<br>(0 to 0)	35.8<br>(8.5 to 86.4)	352<br>(83 to 849)	3<br>(2 to 6)	24.0<br>(11.1 to 47.3)	27<br>(14 to 51)	884%<br>(299 to 2,032%)
**Northern Mariana Islands**	0<br>(0 to 0)	48.5<br>(10.1 to 125.2)	751<br>(157 to 1,940)	2<br>(1 to 6)	7.3<br>(4.1 to 12.5)	9<br>(6 to 15)	433%<br>(108 to 990%)
**Papua New Guinea**	15<br>(4 to 34)	50.8<br>(12.5 to 122.1)	52,914<br>(13,054 to 127,191)	1,797<br>(848 to 3,781)	12,818.5<br>(6,088.8 to 25,246.3)	14,613<br>(7,798 to 26,992)	822%<br>(245 to 1,924%)
**Samoa**	0<br>(0 to 0)	37.8<br>(8.2 to 93.2)	1,043<br>(227 to 2,575)	4<br>(2 to 9)	33.6<br>(14.8 to 67.5)	38<br>(19 to 73)	889%<br>(239 to 2,298%)
**Solomon Islands**	1<br>(0 to 2)	49.7<br>(12.1 to 119.7)	4,074<br>(993 to 9,810)	110<br>(51 to 242)	380.5<br>(197.1 to 675.0)	490<br>(289 to 814)	400%<br>(137 to 877%)
**Tonga**	0<br>(0 to 0)	36.3<br>(8.7 to 86.6)	497<br>(118 to 1,186)	4<br>(2 to 7)	23.2<br>(12.1 to 41.6)	27<br>(16 to 46)	676%<br>(224 to 1,514%)
**Vanuatu**	0<br>(0 to 1)	51.8<br>(12.8 to 122.9)	2,092<br>(515 to 4,963)	44<br>(21 to 87)	268.0<br>(128.2 to 499.5)	312<br>(166 to 547)	695%<br>(220 to 1,548%)

Location	Deaths	Incidence per 1,000	Episodes	Acute DALYs	Undernutrition-associated DALYs	Total DALYs	Percent increase
**North Africa and Middle East**	1,163 (412 to 2,460)	33.6 (13.1 to 80.5)	2,134,468 (831,353 to 5,115,191)	103,990 (37,964 to 217,563)	338,700.8 (194,055.2 to 534,688.9)	486,026 (321,134 to 713,035)	233% (111 to 433%)
**Afghanistan**	59 (2 to 259)	16.0 (1.3 to 91.5)	76,019 (5,939 to 433,878)	14,177 (2,305 to 57,108)	83,117.5 (34,807.1 to 162,216.0)	97,288 (44,560 to 188,091)	1,152% (114 to 4,007%)
**Algeria**	3 (0 to 15)	6.2 (0.5 to 33.8)	31,456 (2,626 to 171,770)	840 (139 to 3,676)	2,742.6 (1,141.4 to 5,473.6)	3,583 (1,574 to 7,592)	664% (59 to 2,215%)
**Bahrain**	0 (0 to 0)	6.0 (0.5 to 37.6)	577 (49 to 3,623)	4 (1 to 14)	33.8 (16.1 to 62.4)	38 (18 to 66)	1,369% (190 to 4,647%)
**Egypt**	966 (396 to 1,856)	132.2 (61.7 to 236.3)	1,580,391 (737,237 to 2,825,424)	98,926 (62,128 to 154,929)	140,645.7 (78,025.2 to 227,536.1)	239,671 (160,243 to 339,357)	150% (71 to 286%)
**Iran**	9 (1 to 25)	15.5 (1.3 to 52.6)	109,904 (9,028 to 372,870)	1,806 (477 to 5,931)	8,088.4 (3,619.6 to 16,663.5)	9,896 (4,838 to 18,580)	672% (118 to 1,936%)
**Iraq**	25 (1 to 118)	10.7 (0.8 to 57.8)	78,882 (5,966 to 428,305)	6,474 (981 to 30,147)	18,704.3 (6,438.0 to 39,022.2)	25,185 (10,069 to 52,792)	598% (50 to 2,299%)
**Jordan**	0 (0 to 1)	6.5 (0.5 to 35.7)	6,198 (509 to 34,254)	93 (17 to 340)	645.0 (277.5 to 1,243.3)	738 (345 to 1,373)	1,234% (158 to 4,192%)
**Kuwait**	0 (0 to 0)	1.7 (0.3 to 7.4)	1,124 (175 to 4,907)	13 (4 to 40)	103.8 (58.8 to 171.1)	117 (70 to 187)	1,203% (214 to 3,107%)
**Lebanon**	0 (0 to 1)	9.5 (0.8 to 54.4)	2,878 (237 to 16,397)	39 (7 to 148)	140.2 (59.2 to 270.1)	180 (81 to 330)	626% (79 to 2,029%)
**Libya**	0 (0 to 1)	3.7 (0.7 to 19.7)	1,828 (334 to 9,716)	38 (9 to 134)	255.7 (134.1 to 433.8)	294 (163 to 490)	1,129% (166 to 3,278%)
**Morocco**	12 (0 to 58)	9.6 (0.8 to 55.7)	21,546 (1,685 to 125,060)	2,984 (470 to 12,873)	5,186.2 (2,280.6 to 10,342.2)	8,173 (3,424 to 19,151)	348% (32 to 1,228%)
**Palestine**	0 (0 to 2)	8.8 (0.7 to 50.3)	9,370 (744 to 53,792)	139 (30 to 465)	604.2 (236.5 to 1,226.4)	744 (318 to 1,416)	711% (102 to 2,328%)
**Oman**	0 (0 to 0)	7.2 (0.6 to 43.0)	3,095 (259 to 18,509)	22 (6 to 71)	136.1 (62.5 to 250.4)	158 (79 to 274)	923% (163 to 2,735%)
**Qatar**	0 (0 to 0)	4.1 (0.3 to 23.7)	514 (43 to 2,998)	6 (1 to 22)	18.3 (8.0 to 35.8)	24 (11 to 46)	544% (76 to 1,828%)
**Saudi Arabia**	0 (0 to 0)	0.6 (0.3 to 0.9)	1,568 (836 to 2,404)	5 (4 to 7)	67.7 (38.2 to 110.5)	73 (43 to 116)	1,384% (707 to 2,410%)
**Sudan**	2 (1 to 5)	1.8 (1.1 to 2.6)	6,804 (3,963 to 9,755)	246 (130 to 484)	21,228.6 (7,947.3 to 43,815.2)	21,474 (8,242 to 44,054)	9,608% (2,903 to 23,419%)
**Syria**	0 (0 to 1)	5.1 (0.4 to 31.2)	9,997 (854 to 60,529)	99 (22 to 333)	917.1 (389.4 to 1,782.0)	1,016 (465 to 1,920)	1,442% (212 to 4,396%)
**Tunisia**	0 (0 to 1)	6.1 (0.5 to 34.8)	5,671 (478 to 32,324)	76 (13 to 307)	302.0 (139.9 to 544.0)	378 (178 to 682)	720% (78 to 2,448%)
**Turkey**	3 (0 to 14)	7.2 (0.6 to 42.5)	44,737 (3,655 to 264,906)	838 (139 to 3,060)	3,003.4 (1,258.7 to 5,870.7)	3,843 (1,715 to 7,441)	716% (74 to 2,588%)
**United Arab Emirates**	0 (0 to 0)	6.6 (0.5 to 39.2)	6,110 (503 to 36,541)	37 (10 to 116)	223.2 (95.1 to 455.8)	260 (123 to 497)	894% (149 to 2,762%)

Location	Deaths	Incidence per 1,000	Episodes	Acute DALYs	Undernutrition-associated DALYs	Total DALYs	Percent increase
**Yemen**	81<br>(2 to 384)	17.6<br>(1.5 to 101.2)	79,427<br>(6,606 to 457,057)	20,577<br>(2,774 to 89,662)	52,537.0<br>(16,899.4 to 115,187.1)	72,893<br>(26,389 to 164,962)	541%<br>(40 to 2,075%)
**South Asia**	3,721<br>(1,221 to 6,898)	23.3<br>(6.5 to 52.5)	3,719,353<br>(1,046,490 to 8,395,037)	326,197<br>(108,319 to 606,171)	839,657.1<br>(612,314.7 to 1,126,177.6)	1,232,870<br>(926,992 to 1,604,822)	217%<br>(123 to 350%)
**Bangladesh**	8<br>(1 to 43)	0.8<br>(0.4 to 2.9)	12,284<br>(6,034 to 42,571)	1,171<br>(330 to 3,309)	25,592.3<br>(12,019.9 to 39,975.9)	26,685<br>(13,319 to 41,426)	3,059%<br>(637 to 8,336%)
**Bhutan**	0<br>(0 to 1)	26.4<br>(1.8 to 125.7)	1,998<br>(134 to 9,507)	55<br>(10 to 193)	557.0<br>(321.0 to 895.5)	612<br>(362 to 963)	1,734%<br>(238 to 5,478%)
**India**	2,297<br>(650 to 4,545)	19.3<br>(4.0 to 48.0)	2,238,807<br>(459,304 to 5,577,011)	251,148<br>(125,449 to 465,843)	526,864.2<br>(377,321.9 to 727,856.5)	778,263<br>(576,672 to 1,067,176)	233%<br>(104 to 444%)
**Nepal**	1<br>(0 to 1)	1.0<br>(0.6 to 1.4)	4,381<br>(2,618 to 6,049)	59<br>(39 to 90)	6,298.3<br>(3,267.0 to 10,078.7)	6,356<br>(3,314 to 10,143)	11,082%<br>(4,996 to 20,181%)
**Pakistan**	1,416<br>(533 to 2,689)	60.1<br>(21.7 to 117.6)	1,469,433<br>(530,782 to 2,874,737)	144,084<br>(81,407 to 237,979)	280,345.3<br>(169,789.8 to 423,680.1)	420,955<br>(264,937 to 579,185)	206%<br>(87 to 389%)
**Sub-Saharan Africa**	42,739<br>(21,904 to 72,248)	213.4<br>(98.8 to 408.9)	33,711,778<br>(15,611,634 to 64,593,983)	3,725,128<br>(1,918,178 to 6,279,192)	6,236,009.0<br>(4,194,672.1 to 8,921,400.5)	10,612,029<br>(8,260,489 to 13,209,260)	139%<br>(90 to 218%)
**Southern Sub-Saharan Africa**	719<br>(294 to 1,295)	103.9<br>(50.6 to 193.7)	941,676<br>(458,361 to 1,755,910)	63,403<br>(26,416 to 113,137)	131,018.5<br>(89,441.9 to 183,119.9)	208,445<br>(159,658 to 274,770)	170%<br>(97 to 274%)
**Botswana**	14<br>(5 to 29)	71.4<br>(23.6 to 168.9)	19,437<br>(6,424 to 45,958)	1,432<br>(817 to 2,507)	1,037.0<br>(483.9 to 1,880.1)	2,470<br>(1,556 to 3,727)	78%<br>(29 to 161%)
**Lesotho**	83<br>(32 to 154)	117.7<br>(41.5 to 259.5)	29,713<br>(10,471 to 65,520)	8,154<br>(4,786 to 13,408)	10,582.4<br>(5,886.3 to 17,642.6)	18,744<br>(12,802 to 26,372)	139%<br>(60 to 260%)
**Namibia**	52<br>(19 to 108)	109.6<br>(35.8 to 248.3)	37,347<br>(12,191 to 84,598)	5,220<br>(2,805 to 9,185)	6,523.5<br>(3,449.0 to 11,505.8)	11,748<br>(7,376 to 17,696)	136%<br>(54 to 277%)
**South Africa**	232<br>(113 to 403)	129.9<br>(69.4 to 224.2)	702,012<br>(375,244 to 1,211,570)	22,829<br>(15,716 to 32,742)	54,873.9<br>(34,574.8 to 84,750.0)	77,726<br>(55,204 to 108,044)	248%<br>(138 to 411%)
**Swaziland**	50<br>(19 to 101)	106.8<br>(36.9 to 249.5)	21,831<br>(7,548 to 51,001)	4,912<br>(2,651 to 8,612)	6,779.9<br>(3,926.5 to 11,139.4)	11,696<br>(8,160 to 16,921)	151%<br>(62 to 300%)
**Zimbabwe**	288<br>(83 to 601)	51.9<br>(10.6 to 128.2)	134,364<br>(27,567 to 331,953)	34,804<br>(15,192 to 77,645)	51,221.7<br>(32,482.5 to 77,565.1)	86,060<br>(56,346 to 134,562)	174%<br>(60 to 378%)
**Western Sub-Saharan Africa**	28,396<br>(13,983 to 50,422)	252.2<br>(109.1 to 492.4)	16,470,528<br>(7,124,982 to 32,155,456)	2,463,110<br>(1,209,737 to 4,360,397)	3,286,075.3<br>(2,123,679.4 to 4,930,228.0)	6,188,684<br>(4,678,178 to 7,955,598)	110%<br>(65 to 188%)
**Benin**	829<br>(223 to 1,772)	195.3<br>(38.0 to 484.5)	381,543<br>(74,234 to 946,426)	90,362<br>(43,066 to 175,933)	74,408.9<br>(34,822.1 to 135,359.0)	164,861<br>(96,884 to 264,776)	93%<br>(31 to 205%)

Location	Deaths	Incidence per 1,000	Episodes	Acute DALYs	Undernutrition-associated DALYs	Total DALYs	Percent increase
**Burkina Faso**	1,675 (857 to 2,989)	562.6 (302.7 to 964.6)	1,810,816 (974,282 to 3,104,984)	161,567 (103,764 to 253,746)	250,037.2 (140,546.9 to 395,001.4)	410,411 (267,627 to 576,093)	161% (71 to 295%)
**Cameroon**	723 (174 to 1,683)	246.7 (42.9 to 615.4)	970,012 (168,665 to 2,420,393)	82,739 (38,490 to 157,151)	151,668.2 (71,179.3 to 276,714.8)	234,490 (135,845 to 376,875)	208% (69 to 481%)
**Cape Verde**	1 (0 to 3)	134.8 (22.4 to 349.8)	10,025 (1,666 to 26,005)	175 (86 to 347)	202.8 (93.4 to 357.7)	378 (229 to 579)	131% (44 to 295%)
**Chad**	1,900 (490 to 4,061)	370.2 (65.7 to 886.1)	954,578 (169,332 to 2,284,561)	209,191 (99,656 to 399,972)	353,931.2 (170,924.7 to 616,751.4)	561,613 (340,892 to 882,150)	190% (64 to 422%)
**Cote d'Ivoire**	1,423 (578 to 2,715)	232.4 (92.0 to 486.3)	812,707 (321,760 to 1,700,429)	141,687 (83,335 to 231,552)	164,090.8 (93,195.3 to 275,816.0)	305,532 (208,067 to 446,680)	124% (53 to 244%)
**The Gambia**	35 (16 to 64)	120.9 (51.7 to 239.0)	44,375 (18,996 to 87,761)	3,443 (2,058 to 5,341)	6,524.2 (4,241.9 to 9,494.1)	9,939 (7,055 to 13,375)	200% (92 to 356%)
**Ghana**	277 (121 to 524)	199.7 (87.3 to 391.1)	921,957 (402,801 to 1,805,100)	28,647 (16,705 to 45,350)	50,723.4 (29,170.7 to 81,983.0)	79,395 (52,135 to 114,334)	188% (88 to 351%)
**Guinea**	395 (102 to 897)	250.8 (44.4 to 611.9)	502,026 (88,906 to 1,224,710)	46,165 (21,986 to 93,755)	89,795.5 (41,171.1 to 157,583.4)	135,872 (77,489 to 216,426)	223% (67 to 508%)
**Guinea-Bissau**	15 (3 to 33)	28.5 (3.2 to 91.9)	8,723 (979 to 28,077)	1,980 (746 to 4,924)	4,132.1 (2,506.4 to 6,415.0)	6,110 (3,912 to 9,260)	262% (77 to 616%)
**Liberia**	332 (82 to 697)	306.5 (54.2 to 754.3)	217,571 (38,481 to 535,497)	36,338 (17,743 to 70,092)	35,807.4 (16,574.1 to 63,277.0)	72,136 (42,069 to 113,957)	111% (35 to 246%)
**Mali**	548 (196 to 1,048)	55.0 (17.6 to 125.1)	169,520 (54,352 to 385,445)	64,273 (30,990 to 142,232)	86,969.3 (55,462.3 to 132,416.5)	150,776 (102,045 to 227,032)	156% (52 to 312%)
**Mauritania**	84 (23 to 181)	236.2 (43.4 to 573.0)	117,878 (21,663 to 286,008)	9,302 (4,424 to 17,507)	11,506.0 (5,683.3 to 19,945.7)	20,817 (12,477 to 32,454)	141% (46 to 330%)
**Niger**	333 (7 to 1,121)	35.1 (1.0 to 142.8)	130,224 (3,771 to 529,753)	87,103 (12,335 to 323,917)	95,483.7 (39,697.5 to 174,572.0)	177,213 (67,109 to 428,280)	212% (0 to 779%)
**Nigeria**	18,861 (9,603 to 33,386)	288.0 (137.8 to 535.8)	8,648,686 (4,139,187 to 16,087,940)	1,806,022 (1,163,446 to 2,783,546)	1,773,842.3 (1,073,902.8 to 2,675,681.8)	3,579,758 (2,565,850 to 4,808,183)	103% (52 to 188%)
**Sao Tome and Principe**	2 (1 to 4)	135.1 (54.3 to 282.8)	4,669 (1,875 to 9,774)	199 (114 to 331)	382.6 (236.0 to 583.0)	582 (406 to 802)	208% (99 to 412%)
**Senegal**	231 (14 to 597)	112.0 (2.5 to 323.6)	287,052 (6,512 to 829,400)	60,317 (8,845 to 245,874)	46,153.8 (19,852.3 to 84,029.2)	106,528 (40,221 to 303,610)	160% (15 to 660%)
**Sierra Leone**	582 (145 to 1,269)	230.2 (43.3 to 575.3)	239,540 (45,077 to 598,765)	64,347 (30,889 to 121,606)	68,218.8 (31,069.1 to 123,918.0)	132,484 (76,389 to 208,829)	120% (40 to 264%)

Location	Deaths	Incidence per 1,000	Episodes	Acute DALYs	Undernutrition-associated DALYs	Total DALYs	Percent increase
**Togo**	153 (38 to 335)	221.7 (40.8 to 543.6)	242,197 (44,548 to 593,910)	17,613 (8,733 to 34,925)	22,196.9 (10,246.2 to 40,620.5)	39,789 (22,768 to 62,869)	144% (43 to 325%)
**Eastern Sub-Saharan Africa**	6,237 (3,079 to 10,409)	112.9 (52.4 to 214.4)	7,043,892 (3,268,919 to 13,374,790)	548,001 (273,263 to 924,229)	1,493,574.0 (1,034,601.1 to 2,030,080.2)	2,156,499 (1,689,633 to 2,743,811)	222% (143 to 321%)
**Burundi**	579 (180 to 1,215)	129.8 (31.1 to 300.2)	274,672 (65,819 to 634,963)	68,042 (31,294 to 146,754)	101,405.2 (54,972.8 to 171,618.1)	168,724 (102,620 to 270,397)	173% (52 to 392%)
**Comoros**	6 (2 to 13)	99.5 (22.5 to 235.6)	9,672 (2,183 to 22,898)	663 (290 to 1,414)	1,814.7 (953.6 to 3,108.6)	2,478 (1,500 to 3,866)	325% (97 to 816%)
**Djibouti**	6 (1 to 15)	94.2 (20.0 to 238.0)	11,812 (2,504 to 29,829)	797 (310 to 1,947)	2,335.2 (1,156.9 to 4,255.7)	3,133 (1,753 to 5,313)	362% (92 to 911%)
**Eritrea**	93 (26 to 199)	108.2 (24.3 to 272.3)	81,212 (18,260 to 204,326)	11,949 (5,064 to 29,291)	18,073.1 (9,852.3 to 29,953.5)	29,824 (18,008 to 48,510)	183% (43 to 415%)
**Ethiopia**	1,143 (483 to 2,219)	91.5 (30.4 to 188.5)	1,351,463 (449,194 to 2,785,144)	116,373 (68,200 to 191,096)	263,050.6 (167,407.0 to 377,863.7)	379,540 (265,170 to 517,325)	242% (118 to 428%)
**Kenya**	124 (17 to 287)	9.7 (1.5 to 29.3)	63,792 (10,207 to 193,187)	15,213 (5,522 to 37,090)	55,470.4 (38,438.9 to 77,177.5)	70,699 (49,319 to 101,003)	456% (142 to 1,030%)
**Madagascar**	857 (262 to 1,798)	120.5 (27.1 to 290.5)	465,347 (104,729 to 1,121,927)	102,763 (47,972 to 219,923)	171,563.6 (91,521.2 to 281,359.9)	271,398 (155,398 to 436,136)	192% (51 to 439%)
**Malawi**	136 (22 to 334)	37.0 (3.9 to 115.9)	119,429 (12,445 to 374,233)	18,217 (6,549 to 41,360)	59,268.5 (34,550.8 to 93,770.5)	77,503 (49,134 to 120,400)	406% (130 to 1,014%)
**Mozambique**	556 (287 to 928)	136.4 (78.1 to 218.7)	684,968 (392,129 to 1,098,386)	53,768 (34,876 to 81,645)	104,404.6 (66,977.7 to 152,602.9)	158,142 (113,172 to 212,213)	203% (105 to 343%)
**Rwanda**	3 (1 to 18)	1.2 (0.6 to 4.2)	2,179 (1,088 to 7,707)	397 (117 to 1,259)	11,310.4 (6,500.6 to 18,779.6)	11,707 (6,750 to 19,250)	4,174% (776 to 11,089%)
**Somalia**	154 (41 to 369)	120.7 (29.6 to 269.8)	142,714 (35,034 to 319,039)	19,543 (8,551 to 44,818)	56,974.1 (30,802.3 to 95,462.0)	76,396 (46,146 to 124,615)	350% (106 to 752%)
**South Sudan**	279 (71 to 646)	137.3 (30.5 to 335.3)	409,411 (90,910 to 999,480)	35,604 (14,604 to 78,459)	156,386.8 (80,499.0 to 254,301.7)	191,942 (113,602 to 293,500)	525% (156 to 1,226%)
**Tanzania**	544 (278 to 915)	77.4 (39.9 to 136.7)	726,466 (375,057 to 1,283,681)	52,969 (34,008 to 80,515)	130,173.4 (82,840.6 to 189,708.3)	183,195 (130,719 to 248,605)	257% (135 to 441%)
**Uganda**	1,253 (666 to 2,160)	271.3 (135.0 to 478.1)	2,047,638 (1,018,964 to 3,608,333)	121,110 (77,782 to 182,305)	249,877.0 (165,579.2 to 367,602.7)	371,080 (274,282 to 498,835)	215% (115 to 365%)

Location	Deaths	Incidence per 1,000	Episodes	Acute DALYs	Undernutrition-associated DALYs	Total DALYs	Percent increase
**Zambia**	505 (258 to 890)	239.6 (123.1 to 407.7)	676,146 (347,389 to 1,150,883)	49,222 (31,454 to 77,549)	111,466.4 (69,275.2 to 167,766.4)	160,737 (111,417 to 221,373)	238% (122 to 416%)
**Central Sub-Saharan Africa**	7,387 (3,599 to 13,036)	439.7 (205.1 to 841.2)	9,335,506 (4,353,774 to 17,858,382)	650,614 (320,586 to 1,139,678)	1,325,341.1 (777,649.3 to 2,119,147.4)	2,058,400 (1,426,990 to 2,877,238)	177% (90 to 336%)
**Angola**	2,490 (1,175 to 4,558)	598.3 (310.6 to 1,075.9)	2,992,584 (1,553,468 to 5,381,845)	239,836 (147,675 to 378,066)	334,097.8 (189,426.4 to 530,470.1)	573,110 (385,889 to 812,527)	147% (66 to 273%)
**Central African Republic**	353 (160 to 661)	242.0 (106.6 to 468.1)	191,436 (84,309 to 370,261)	34,159 (20,626 to 54,558)	95,429.4 (54,186.8 to 152,887.0)	129,505 (86,065 to 188,087)	296% (135 to 558%)
**Congo**	152 (62 to 307)	383.9 (156 to 785.0)	286,035 (116,909 to 584,957)	15,769 (8,498 to 26,523)	26,999.0 (14,162.4 to 47,844.8)	42,784 (26,599 to 66,287)	186% (74 to 402%)
**Democratic Republic of the Congo**	4,362 (1,687 to 8,999)	400.4 (166.1 to 804.2)	5,743,449 (2,383,552 to 11,536,796)	441,111 (249,041 to 743,734)	861,926.1 (392,576.5 to 1,574,291.1)	1,302,924 (782,583 to 1,995,920)	212% (77 to 456%)
**Equatorial Guinea**	3 (1 to 7)	380.6 (149.1 to 790.0)	37,337 (14,627 to 77,505)	373 (219 to 623)	1,725.0 (838.1 to 3,162.6)	2,099 (1,215 to 3,580)	496% (185 to 1,076%)
**Gabon**	27 (11 to 54)	334.3 (135.8 to 676.1)	83,003 (33,711 to 167,889)	2,812 (1,531 to 4,979)	5,163.7 (2,667.4 to 8,784.7)	7,978 (5,058 to 11,976)	200% (76 to 416%)

References

1 GBD Diarrhoeal Diseases Collaborators. Estimates of global, regional, and national morbidity, mortality, and aetiologies of diarrhoeal diseases: a systematic analysis for the Global Burden of Disease Study 2015. *Lancet Infect Dis* 2017; published online June 1. DOI:10.1016/S1473-3099(17)30276-1.

2 GBD 2016 Causes of Death Collaborators. Global, regional, and national age-sex specific mortality for 264 causes of death, 1980-2016: a systematic analysis for the Global Burden of Disease Study 2016. *Lancet* 2017; **390**: 1151–210.

3 GBD 2016 Disease and Injury Incidence and Prevalence Collaborators. Global, regional, and national incidence, prevalence, and years lived with disability for 328 diseases and injuries for 195 countries, 1990-2016: a systematic analysis for the Global Burden of Disease Study 2016. *Lancet* 2017; **390**: 1211–59.

4 GBD 2016 Risk Factors Collaborators. Global, regional, and national comparative risk assessment of 84 behavioural, environmental and occupational, and metabolic risks or clusters of risks, 1990-2016: a systematic analysis for the Global Burden of Disease Study 2016. *Lancet* 2017; **390**: 1345–422.

5 Foreman KJ, Lozano R, Lopez AD, Murray CJ. Modeling causes of death: an integrated approach using CODEm. *Popul Health Metr* 2012; **10**: 1.

6 GBD 2016 Disease and Injury Incidence and Prevalence Collaborators. Global, regional, and national incidence, prevalence, and years lived with disability for 328 diseases and injuries for 195 countries, 1990-2016: a systematic analysis for the Global Burden of Disease Study 2016. *Lancet Lond Engl* 2017; **390**: 1211–59.

7 Miettinen OS. Proportion of disease caused or prevented by a given exposure, trait or intervention. *Am J Epidemiol* 1974; **99**: 325–32.

8 Kotloff KL, Nataro JP, Blackwelder WC, *et al.* Burden and aetiology of diarrhoeal disease in infants and young children in developing countries (the Global Enteric Multicenter Study, GEMS): a prospective, case-control study. *Lancet* 2013; **382**: 209–22.

9 Liu J, Platts-Mills JA, Juma J, *et al.* Use of quantitative molecular diagnostic methods to identify causes of diarrhoea in children: a reanalysis of the GEMS case-control study. *Lancet Lond Engl* 2016; **388**: 1291–301.

10 World Health Organization. WHO Child Growth Standards: Methods and development. 2006 http://www.who.int/childgrowth/standards/technical_report/en/ (accessed July 27, 2016).

11 Kuczmarski R, Ogden C, Grummer-Strawn L, et al. CDC Growth Charts: United States. Advance data from vital and health statistics. Hyattsville, Maryland: National Center for Health Statistics http://www.cdc.gov/nchs/data/ad/ad314.pdf (accessed Aug 23, 2016).

12 Agnew DG, Lima AA, Newman RD, *et al.* Cryptosporidiosis in northeastern Brazilian children: association with increased diarrhea morbidity. *J Infect Dis* 1998; **177**: 754–60.

13 Checkley W, Epstein LD, Gilman RH, Black RE, Cabrera L, Sterling CR. Effects of Cryptosporidium parvum infection in Peruvian children: growth faltering and subsequent catch-up growth. *Am J Epidemiol* 1998; **148**: 497–506.

14 Checkley W, Gilman RH, Epstein LD, *et al.* Asymptomatic and symptomatic cryptosporidiosis: their acute effect on weight gain in Peruvian children. *Am J Epidemiol* 1997; **145**: 156–63.

15 Sarkar R, Tate JE, Ajjampur SSR, *et al.* Burden of diarrhea, hospitalization and mortality due to cryptosporidial infections in Indian children. *PLoS Negl Trop Dis* 2014; **8**: e3042.

16 Korpe PS, Haque R, Gilchrist C, *et al.* Natural History of Cryptosporidiosis in a Longitudinal Study of Slum-Dwelling Bangladeshi Children: Association with Severe Malnutrition. *PLoS Negl Trop Dis* 2016; **10**: e0004564.

17 Sow SO, Muhsen K, Nasrin D, *et al.* The Burden of Cryptosporidium Diarrheal Disease among Children < 24 Months of Age in Moderate/High Mortality Regions of Sub-Saharan Africa and South Asia, Utilizing Data from the Global Enteric Multicenter Study (GEMS). *PLoS Negl Trop Dis* 2016; **10**: e0004729.

18 Lima AA, Moore SR, Barboza MS, *et al.* Persistent diarrhea signals a critical period of increased diarrhea burdens and nutritional shortfalls: a prospective cohort study among children in northeastern Brazil. *J Infect Dis* 2000; **181**: 1643–51.

19 MAL-ED Network Investigators. The MAL-ED study: a multinational and multidisciplinary approach to understand the relationship between enteric pathogens, malnutrition, gut physiology, physical growth, cognitive development, and immune responses in infants and children up to 2 years of age in resource-poor environments. *Clin Infect Dis Off Publ Infect Dis Soc Am* 2014; **59 Suppl 4**: S193-206.

20 Mølbak K, Andersen M, Aaby P, *et al.* Cryptosporidium infection in infancy as a cause of malnutrition: a community study from Guinea-Bissau, west Africa. *Am J Clin Nutr* 1997; **65**: 149–52.

21 Moore SR, Lima NL, Soares AM, *et al.* Prolonged episodes of acute diarrhea reduce growth and increase risk of persistent diarrhea in children. *Gastroenterology* 2010; **139**: 1156–64.

22 Kirkpatrick BD, Colgate ER, Mychaleckyj JC, *et al.* The 'Performance of Rotavirus and Oral Polio Vaccines in Developing Countries' (PROVIDE) study: description of methods of an interventional study designed to explore complex biologic problems. *Am J Trop Med Hyg* 2015; **92**: 744–51.

23 Altman DG, Bland JM. How to obtain the confidence interval from a P value. *BMJ* 2011; **343**: d2090.

24 Olofin I, McDonald CM, Ezzati M, *et al.* Associations of suboptimal growth with all-cause and cause-specific mortality in children under five years: a pooled analysis of ten prospective studies. *PloS One* 2013; **8**: e64636.

25 GBD 2016 Risk Factors Collaborators. Global, regional, and national comparative risk assessment of 84 behavioural, environmental and occupational, and metabolic risks or clusters of risks, 1990-2016: a systematic analysis for the Global Burden of Disease Study 2016. *Lancet Lond Engl* 2017; **390**: 1345–422.

26 Troeger C, Colombara DV, Rao PC, *et al.* Global disability-adjusted life-year estimates of long-term health burden and undernutrition attributable to diarrhoeal diseases in children younger than 5 years. *Lancet Glob Health* 2018; **6**: e255–69.

27 GBD 2016 Disease and Injury Incidence and Prevalence Collaborators. Global, regional, and national incidence, prevalence, and years lived with disability for 328 diseases and injuries for 195 countries, 1990-2016: a systematic analysis for the Global Burden of Disease Study 2016. *Lancet Lond Engl* 2017; **390**: 1211–59.

28 GBD 2016 Causes of Death Collaborators. Global, regional, and national age-sex specific mortality for 264 causes of death, 1980-2016: a systematic analysis for the Global Burden of Disease Study 2016. *Lancet Lond Engl* 2017; **390**.
